# Pharmacologic LDH inhibition redirects intratumoral glucose uptake and improves antitumor immunity in solid tumor models

**DOI:** 10.1172/JCI177606

**Published:** 2024-09-03

**Authors:** Svena Verma, Sadna Budhu, Inna Serganova, Lauren Dong, Levi M. Mangarin, Jonathan F. Khan, Mamadou A. Bah, Anais Assouvie, Yacine Marouf, Isabell Schulze, Roberta Zappasodi, Jedd D. Wolchok, Taha Merghoub

**Affiliations:** 1 Pharmacology Program,; 2 Swim Across America, and Ludwig Collaborative Laboratory, Department of Pharmacology,; 3 Sandra and Edward Meyer Cancer Center,; 4 Department of Medicine,; 5 Immunology and Microbial Pathogenesis Program,; 6 Parker Institute for Cancer Immunotherapy, Weill Cornell Medicine, New York, New York, USA.

**Keywords:** Immunology, Metabolism, Cancer immunotherapy, Glucose metabolism, Pharmacology

## Abstract

Tumor reliance on glycolysis is a hallmark of cancer. Immunotherapy is more effective in controlling glycolysis-low tumors lacking lactate dehydrogenase (LDH) due to reduced tumor lactate efflux and enhanced glucose availability within the tumor microenvironment (TME). LDH inhibitors (LDHi) reduce glucose uptake and tumor growth in preclinical models, but their impact on tumor-infiltrating T cells is not fully elucidated. Tumor cells have higher basal LDH expression and glycolysis levels compared with infiltrating T cells, creating a therapeutic opportunity for tumor-specific targeting of glycolysis. We demonstrate that LDHi treatment (a) decreases tumor cell glucose uptake, expression of the glucose transporter GLUT1, and tumor cell proliferation while (b) increasing glucose uptake, GLUT1 expression, and proliferation of tumor-infiltrating T cells. Accordingly, increasing glucose availability in the microenvironment via LDH inhibition leads to improved tumor-killing T cell function and impaired Treg immunosuppressive activity in vitro. Moreover, combining LDH inhibition with immune checkpoint blockade therapy effectively controls murine melanoma and colon cancer progression by promoting effector T cell infiltration and activation while destabilizing Tregs. Our results establish LDH inhibition as an effective strategy for rebalancing glucose availability for T cells within the TME, which can enhance T cell function and antitumor immunity.

## Abstract 2

Pharmacologic LDH inhibition redirects intratumoral glucose consumption within the tumor microenvironment to favor antitumor T cell function and improve the efficacy of immune checkpoint blockade.

## Graphical abstract

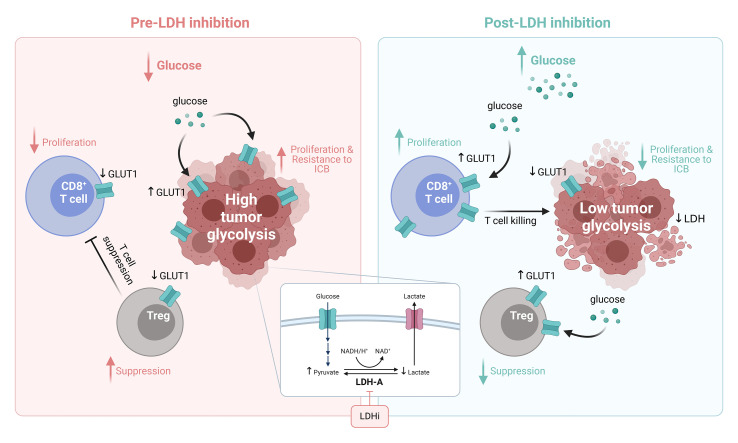

## Introduction

The Warburg effect describes the propensity of cancer cells to favor the conversion of glucose to lactate for energy production, even in the presence of oxygen (1, 2). In this process, glucose is rapidly consumed and converted to pyruvate, which is then diverted from oxidative phosphorylation to lactate production through the activity of the enzyme lactate dehydrogenase (LDH) (3, 4). Over the last century, scientists have expanded upon this phenomenon and deemed altered tumor metabolism a major hallmark of cancer (5, 6). Preferential engagement in glycolysis contributes to the growth, progression, and metastasis of many tumor types, including melanoma, colon cancer, and triple-negative breast cancer (TNBC). Therefore, glycolysis constitutes an attractive pathway to target in cancer therapy (6–9). Tumor cells’ reliance on glycolysis is also a well-documented mechanism of resistance to immunotherapy, including immune checkpoint blockade (ICB) (8, 10–12). Antitumor immune cell activity within the tumor is affected by lactate-mediated acidification. In addition, immune cells in the tumor microenvironment (TME) must compete for glucose with glycolytic tumor cells (13–15).

Several studies have advocated for targeting glycolytic proteins in combination with immunotherapy (10, 13–19). A substantial challenge in exploiting cancer metabolism as a therapeutic target is achieving tumor-suppressing effects while simultaneously minimizing harm to healthy cells (17, 20, 21). For example, the glucose analog 2-DG has been proposed in both experimental and clinical oncology as a potential pharmacological agent for targeting glucose metabolism. However, the clinical application of 2-DG has been limited due to its lack of specificity and undesirable side effects in healthy tissues (20). Since noncancerous cells, such as immune cells, need to metabolize nutrients as part of regular physiologic function, there is a critical need to elucidate metabolic targets differentially used by cancer cells that can be exploited in immune-based combination therapy.

Here, we describe LDH as a rational antitumor target for combination with immunotherapy. LDH is a cytoplasmic enzyme consisting of 4 subunits of LDHA and/or LDHB, differently assorted depending on the tissue type. While LDHB is predominantly expressed in the heart, brain, and kidney, LDHA is primarily expressed in skeletal and liver tissue and is widely expressed in malignancies as well (22, 23). The LDHA subunit has a higher affinity for pyruvate and therefore favors the conversion of pyruvate to lactate, sustaining glycolysis, while LDHB preferentially converts lactate to pyruvate (22, 24). Genetic dampening of tumor LDHA decreases glucose consumption and lactate production by tumor cells, and this was associated with slower tumor progression, higher CD8^+^ T cell infiltration, and improved overall survival in preclinical models of various solid tumors (11, 13, 25, 26). Additionally, heightened levels of LDH in the bloodstream have been traditionally regarded as an indicator of unfavorable prognosis for patients with various cancer types, including lymphoma (27, 28), melanoma (29), and lung cancer (30). High serum LDH has been typically linked to increased tumor burden and poorer survival (3, 31, 32). Serum LDH can also be a predictive biomarker of poor responses to immunotherapies (12, 33).

LDH inhibitors (LDHis), such as GNE-140, have been reported to inhibit tumor glycolysis and slow tumor cell proliferation in preclinical models (34). However, these inhibitors have yet to advance to clinical trials (17, 21), and preclinical antitumor efficacy in combination with immunotherapy and corresponding mechanisms of action has not been comprehensively assessed. Specifically, the optimal combination of LDHi with ICB to maximize antitumor immune and therapeutic responses is unknown. As immune cells also need to engage in glycolysis to execute their tumoricidal function (35–37), there is a critical need to determine appropriate regimens and schedules for use of LDHi in combination with ICB to preferentially dampen glycolysis in tumor cells without affecting immune cell functionality for successful therapeutic outcomes.

Our group has recently reported that the efficacy of CTLA-4 blockade is increased in the setting of glycolysis-low LDHA-knockdown (LDHA-KD) tumors. In these tumors, we found that Tregs become unstable and can convert into IFN-γ–producing effector-like T cells when CTLA-4 is inhibited (25, 38, 39). In line with our prior data, Treg phenotype and function were found to be supported in the presence of lactic acid and destabilized in high concentrations of glucose (25, 40, 41). Based upon these observations, we sought to determine whether resistance to CTLA-4 blockade in highly glycolytic cancers may be blunted using glycolysis inhibitors, such as LDHis. Specifically, we reasoned that this strategy could reverse the high lactate-to-glucose ratio within tumors and destabilize tumor metabolism and Tregs, leading to enhanced therapeutic activity when combined with CTLA-4 blockade, based on our prior findings in genetically modified tumor models.

In this study, we report that systemic LDH inhibition using GNE-140 reduces glucose uptake in tumor cells, increases glucose availability within the TME, and subsequently increases T cell glucose uptake. In turn, LDHi improves the response to ICB in preclinical models of melanoma and colon cancer. We show that increasing glucose availability within the TME improves antitumor T cell killing and can also blunt Treg suppression, further confirming that glucose availability in the TME is a determinant for antitumor immunity. Our results establish the rationale for testing the combination of ICB with inhibitors of glycolysis for the treatment of highly glycolytic cancers.

## Results

### LDH is a rational target for immunotherapy combinations.

**Figure 1:**
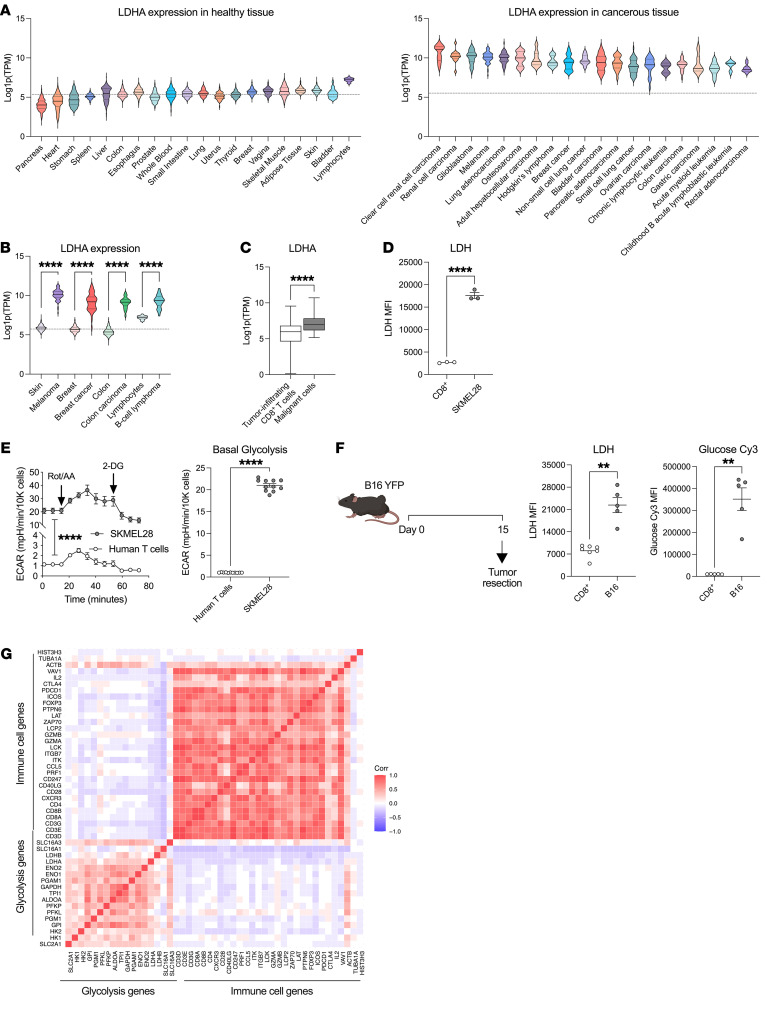
Differential expression of LDHA in tumor cells compared with T cells. (**A**) Average LDHA RNA expression across the indicated human tissue types; lines on graph denote median gene expression in healthy tissue. Source: CCLE (*n* = 150) and GTEx (*n* = 80). (**B**) Comparison in LDHA RNA expression between the indicated normal and cancerous cell types of interest. (**C**) Quantified LDHA RNA expression from tumor-infiltrating CD8^+^ T cells versus malignant cells from human melanoma scRNA-Seq data. Source: Tirosh et al. (42) (*n* = 19). (**D**) Flow cytometry quantification of intracellular LDH (*n* = 3) and (**E**) ECAR by Seahorse in cultured SK-MEL-28 melanoma cells and activated T cells isolated from healthy donors. T cells were analyzed on day 3 after activation with anti-CD3/CD28 beads. Data show 1 representative experiment of 3 independent experiments (*n* = 12). (**F**) Flow cytometry quantification of intracellular LDH MFI and glucose-Cy3 MFI in tumor-infiltrating CD8^+^ T cells and tumor cells from established B16-YFP murine tumors as indicated in the schematic. Data show 1 representative experiment of 3 independent experiments (*n* = 6). (**G**) Correlation matrix between expression of the indicated genes related to immune cell activation and glycolysis in human melanoma cases from the TCGA (*n* = 400). All statistics produced by unpaired *t* tests with Welch’s correction implemented in GraphPad Prism. **P < 0.01; ****P < 0.0001. Data are represented as mean ± SEM.

In both aerobic and anaerobic states, glucose is converted to pyruvate. In aerobic conditions, pyruvate enters the citric acid cycle and undergoes oxidative phosphorylation. Under conditions of low oxygen availability in tissues, pyruvate is redirected away from mitochondria and converted into lactate through the reaction catalyzed by LDH. While energetically less favorable, glycolysis with lactate production is the preferred pathway in cancer cells, as it sustains their main anabolic processes (2, 4). By analyzing human tissue sample data from the Cancer Cell Line Encyclopedia (CCLE) and Genotype-Tissue Expression (GTEx) sources, we found that LDHA is highly expressed in many cancers as compared with normal tissues (Figure 1A). Because activated lymphocytes also upregulate LDHA and engage in glycolysis in order to execute tumoricidal function (37), we compared LDHA expression levels in human lymphocytes to that of cancer types and corresponding normal tissue. We found that while LDHA levels are slightly higher in activated lymphocytes compared with other normal tissues, LDHA levels in glycolytic malignant tissues, such as melanoma, colorectal cancer, breast cancer, and B cell lymphoma, are significantly higher (Figure 1B). Upon reanalyzing single-cell RNA-Seq (scRNA-Seq) data from human melanoma samples (42), we confirmed that malignant cells within the tumor overexpress LDHA compared with CD8^+^ tumor-infiltrating lymphocytes (TILs) (Figure 1C). In addition, human melanoma cells (SK-MEL-28) cultured in vitro showed significantly higher intracellular LDH, extracellular acidification rate (ECAR), and basal levels of glycolysis compared with αCD3/αCD28-activated human T cells (Figure 1, D and E).

To corroborate these findings in vivo, we implanted the murine melanoma cell line B16-F10 (B16) tagged with fluorescent protein YFP orthotopically in mice and compared LDH expression and glucose uptake potential between B16-YFP tumor cells and CD8^+^ TILs by flow cytometry after 2 weeks of tumor progression (Supplemental Figure 1). We found that B16 tumor cells have greater levels of intracellular LDH and glucose uptake, based on cellular uptake of the fluorescent glucose tracker glucose-Cy3, than CD8^+^ T cells from the same tumors (Figure 1F). Therefore, tumor cells retain a significantly higher LDH expression level and overall glycolytic program compared with activated CD8^+^ T cells, creating a therapeutic opportunity for preferential targeting of this enzyme in tumor cells over immune cells.

Serum LDH is a negative prognostic factor for many cancers (3, 12, 27–30, 33). However, its relationship with LDH expression in tumor tissue in association with response to immunotherapy is unknown. We found serum LDH positively correlates with tumor LDH activity as well as tumor volume 10 and 20 days after B16 implantation in immunocompetent mice (Supplemental Figure 2, A and B). Consistently, we found elevated LDHA expression in human melanoma samples from TCGA is associated with poorer survival (Supplemental Figure 2C). We previously demonstrated that the expression of genes involved in glucose metabolism, including LDHA, negatively correlates with immune cell infiltration in a small cohort of patients with melanoma (25). We thus investigated this feature across human melanoma specimens in the TCGA, and consistently observed an inverse relationship between glycolysis genes and genes related to immune cell activation (Figure 1G), suggesting that tumor glycolysis may negatively condition the tumor immune microenvironment and confer resistance to immunotherapy (43). Taken together, these data provide a strong rationale for testing pharmacologic inhibition of LDHA to counteract tumor glycolysis and improve antitumor T cell function.

### The antitumor activity of LDHi is dependent on tumor expression of LDHA and the adaptive immune system.

**Figure 2:**
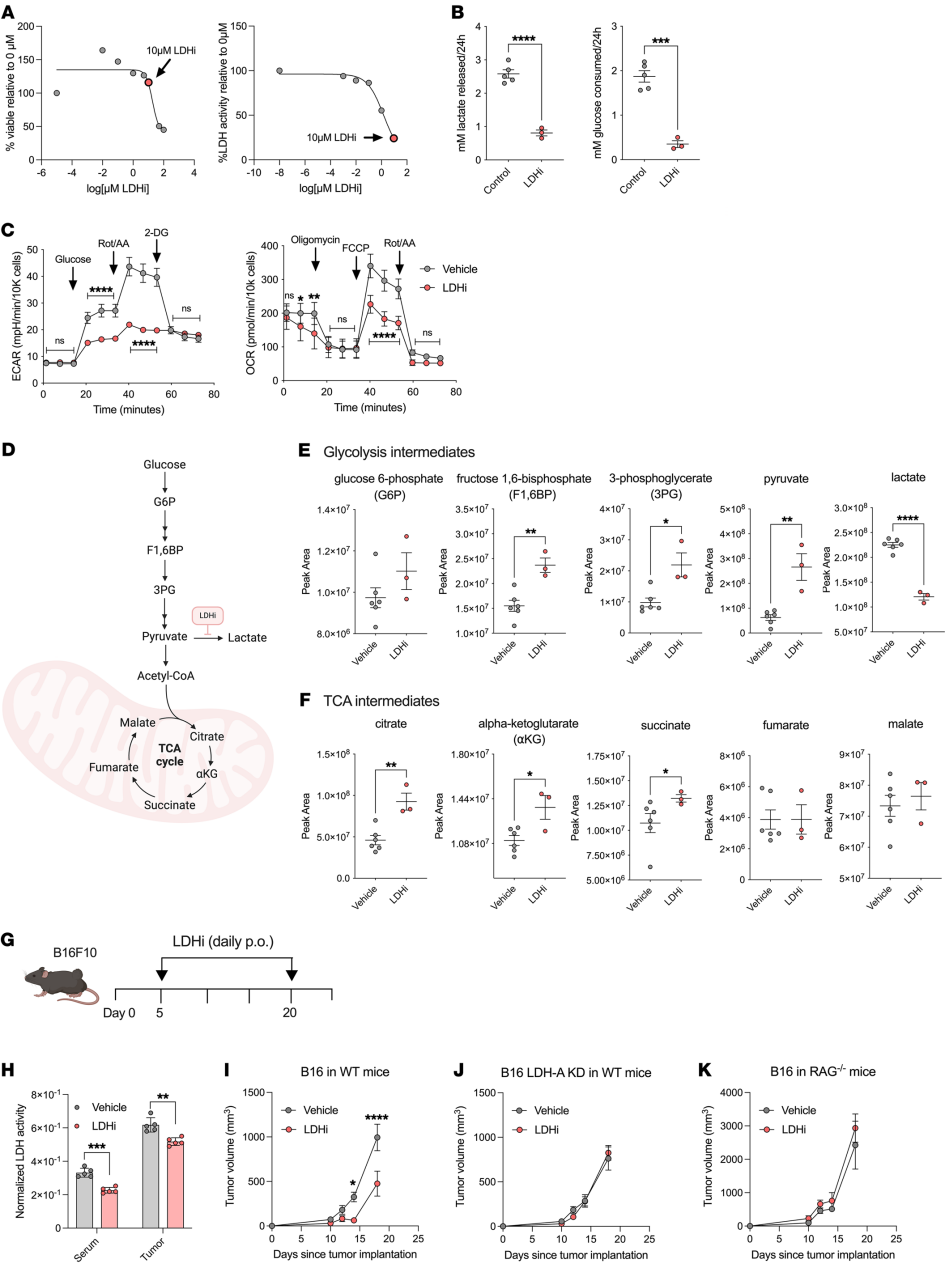
LDHi reduces tumor glycolysis and progression. (**A**) B16 viability and LDH activity 24 hours after treatment with GNE-140 (LDHi) at the indicated concentrations (*n* = 6). (**B**) Extracellular lactate and glucose from B16 cells treated with 10 μM LDHi or control vehicle for 24 hours, normalized by cell number (*n* = 3–5). (**C**) Extracellular acidification (ECAR) and OCRs of B16 cells treated with 10 μM LDHi or vehicle control for 24 hours, measured by Seahorse assays (glycolysis stress test and mitochondrial stress test). Data are normalized by cell number (*n* = 10). (**D**–**F**) Intracellular glycolysis. (**E** and **F**) TCA metabolites quantified by liquid chromatography–mass spectrometry (LC-MS) from B16 whole-cell lysates treated with 10 μM LDHi or vehicle control for 24 hours (*n* = 3–6). (**G** and **H**) Quantification of serum and tumor LDH activity from B16-bearing mice treated with 100 mg/kg LDHi or vehicle control (daily, p.o.) for 2 weeks, as indicated in the schematic (*n* = 5 mice/group). Sera and tumor lactate and LDH activity were analyzed 24 hours after the last treatment with LDHi. (**I**–**K**) Tumor growth curves of B16 or B16 LDHA KD in the indicated mouse strains treated with 100 mg/kg LDHi or vehicle control (daily, p.o.) for 2 weeks as indicated in the schematic (*n* = 10 mice/group). All data show 1 representative experiment of 3 independent experiments. All statistics produced by (**B**, **E**, and **F**) unpaired *t* tests with Welch’s correction or (**C** and **I**) 2-way ANOVA with Bonferroni’s multiple-comparisons test implemented in GraphPad Prism. *P < 0.05; **P < 0.01; ****P < 0.0001. Data are represented as mean ± SEM.

Small-molecule inhibitors of the key glycolytic enzyme LDH have been proposed for cancer treatment to disable tumor dependence on glycolysis and addiction to glucose (16, 44, 45). However, there is a lack of mechanistic understanding in terms of how specifically targeting glycolysis can potentiate immunotherapies. To address this gap, we used the potent LDHA/B inhibitor GNE-140 (34) in our solid tumor model systems. By in vitro treatment with GNE-140 at increasing concentrations, we showed that LDH activity was significantly inhibited at sublethal concentrations (Figure 2A). LDHi also conferred a decrease in overall glycolytic flux, including lactate production, glucose consumption, and ECAR, in B16 cells upon treatment in vitro (Figure 2, B and C). Two treatments with LDHi 24 hours apart were sufficient to reduce LDHA protein levels in B16 cells (Supplemental Figure 3A). We also observed compensatory metabolic rewiring in B16 cells upon LDHi treatment through increases in oxygen consumption rate (OCR) (Figure 2C). Consistently, LDHi administration led to a decrease in intracellular lactate and an increase in intracellular pyruvate and citric acid cycle (TCA) intermediates in B16 cells, indicating a shift from glycolysis to mitochondrial metabolism (Figure 2, D–F).

We next examined the effects of systemic LDHi treatment in vivo and found decreased serum lactate and LDH activity as well as tumor LDH activity after 2 weeks of daily LDHi administration in B16-bearing mice (Figure 2, G and H, and Supplemental Figure 3B). We observed that systemic LDH inhibition delays B16 melanoma growth in vivo (Figure 2I), and we confirmed these results in another glycolytic tumor model — MC38 colorectal cancer (Supplemental Figure 3C). To interrogate the target specificity of this treatment, we tested the effects of this LDHi in mice implanted with LDHA-KD B16 cells. Without canonical expression of LDHA, LDHi failed to control melanoma growth in mice (Figure 2J). Notably, LDHi did not slow tumor growth in RAG-deficient mice, which lack an adaptive immune system, despite producing the same decrease in serum lactate levels (Figure 2K and Supplemental Figure 3B). Therefore, tumor cell expression of LDHA as well as the adaptive immune system are both essential for the antitumor effect of LDHA inhibition.

### Tumor cells display more glycolytic sensitivity to LDH inhibition than immune cells.

**Figure 3:**
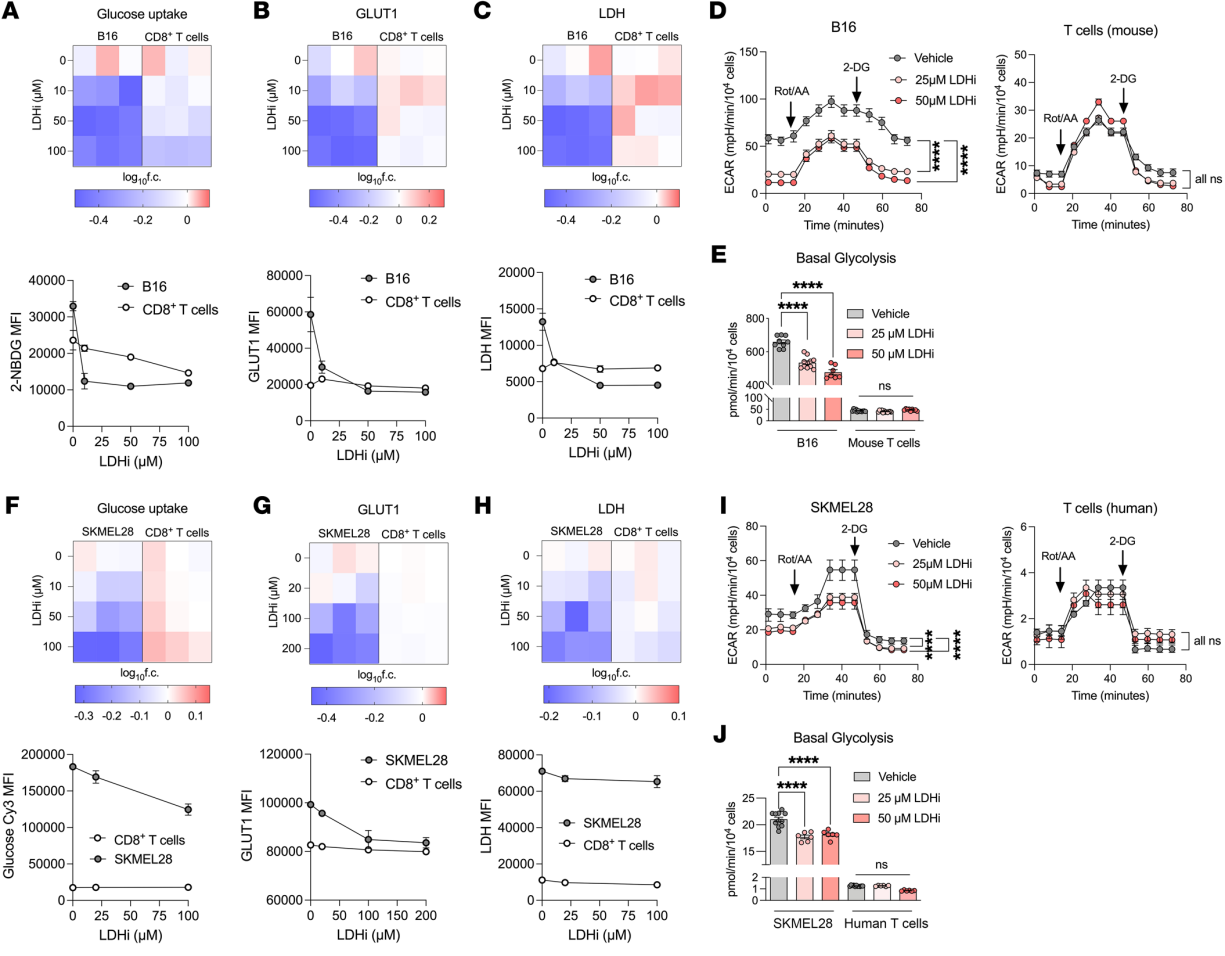
Tumor cells display greater glycolytic sensitivity to LDH inhibition than immune cells. (**A**–**C**) Normalized fold change and absolute flow cytometry quantifications of (**A**) 2-NBDG, (**B**) GLUT1, and (**C**) LDH MFIs in B16 cells and activated mouse CD8^+^ T cells treated with increasing concentrations of LDHi relative to vehicle in vitro. Mouse T cells were treated with LDHi 24 hours after aCD3/aCD28 activation and analyzed 24 hours later. (**D** and **E**) ECARs and (**D** and **E**) basal glycolysis normalized by cell number of B16 cells and activated mouse CD8^+^ T cells treated in vitro with LDHi at the indicated concentrations or vehicle as in **A**–**C**. (**F**–**H**) Normalized fold change and absolute flow cytometry quantifications of (**F**) 2-NBDG, (**G**) GLUT1, and (**H**) LDH MFIs of SK-MEL-28 cells and activated human CD8^+^ T cells from a representative healthy donor treated with increasing concentrations of LDHi relative to vehicle in vitro. Human T cells were treated with LDHi 48 hours after αCD3/αCD28 activation and analyzed 24 hours later. (**I** and **J**) ECAR and (**I** and **J**) basal glycolysis normalized by cell numbers of SK-MEL-28 cells and activated human CD8^+^ T cells treated with LDHi at the indicated concentrations or vehicle in vitro as in **F**–**H**. Data show 1 representative experiment of 3 independent experiments (*n* = 3–4 technical replicates for flow experiments, 9–12 technical replicates for Seahorse experiments). All statistics produced by 2-way ANOVA with Bonferroni’s multiple-comparisons test implemented in GraphPad Prism. ****P < 0.0001. Data are represented as mean ± SEM.

Since the adaptive immune system is required for LDH inhibition to delay tumor growth, we sought to test the effects of LDHi on immune cells. Upon activation, T cells increase their rates of glycolysis and glucose uptake to perform effector functions (35–37); therefore, we investigated how LDHi affects glycolytic parameters in activated T cells compared with tumor cells. To interrogate this, we assessed glucose uptake based on 2-NBDG and glucose-Cy3 as well as GLUT1 and LDH expression on both tumor and activated T cells in isolation upon treatment with LDHi in vitro by flow cytometry. We observed a marked decrease in B16 melanoma cell glucose uptake, GLUT1, and LDH expression upon treatment with increasing concentrations of LDHi (Figure 3, A–C, and Supplemental Figure 4A). Interestingly, activated T cells did not respond as dramatically to LDH inhibition by the same glycolytic parameters (Figure 3, A–C, and Supplemental Figure 4A). In accordance with these data, when assessing live cell metabolism upon treatment with LDHi, B16 cells displayed significantly lower ECAR and basal levels of glycolysis (Figure 3, D and E). Activated T cells, however, were not as sensitive to the same doses of LDHi and were able to maintain their ECAR upon LDH inhibition (Figure 3, D and E). These trends were confirmed in human melanoma cell line SK-MEL-28 and activated T cells from healthy donors (Figure 3, F–J, and Supplemental Figure 4B). Both human and mouse melanoma tumor cells and T cells slightly increased their OCR and maximal levels of respiration upon LDH inhibition, while higher doses of LDHi reduced ATP production in tumor cells and not T cells (Supplemental Figure 4, C and D). Additionally, when we analyzed tumor and CD8^+^ T cells from untreated B16 tumors after 2 weeks of progression ex vivo, Ki67 expression strongly correlated with LDH and GLUT1 expression in B16 tumor cells, but this correlation was less apparent in CD8^+^ TILs, suggesting that the glycolytic markers LDH and GLUT1 may be more important for tumor cell proliferation than T cell proliferation (Supplemental Figure 4E).

### LDH inhibition shifts the glycolytic balance between tumor and infiltrating T cells.

**Figure 4:**
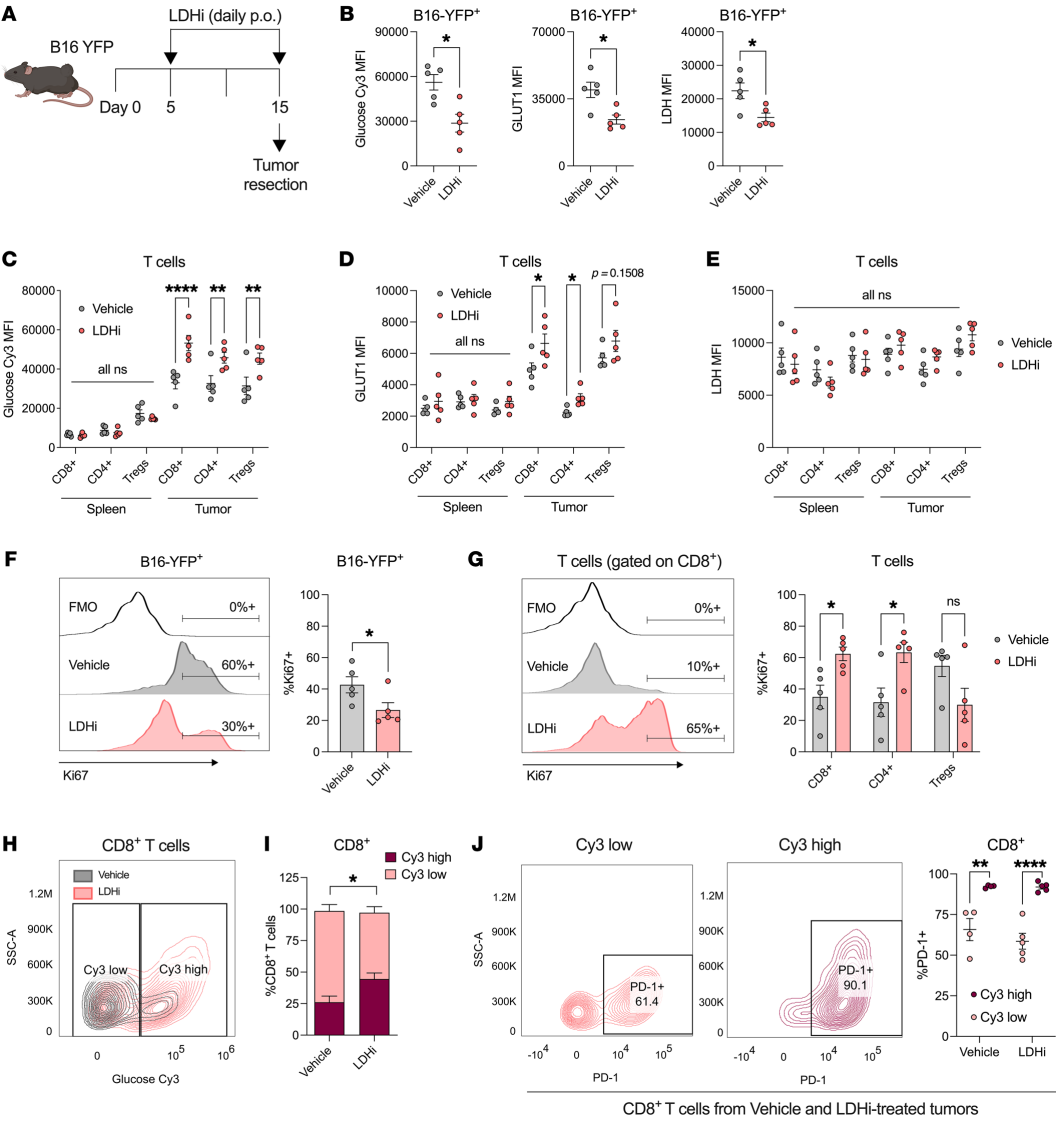
Differential effects of LDH inhibition in tumor cells compared with tumor-infiltrating T cells. (**A**) Mice (*n* = 5/group) were implanted with B16-YFP cells and treated with LDHi (100 mg/kg) or vehicle control as indicated in the schematic (**A**). Tumors were processed for flow cytometry quantification of glucose-Cy3, GLUT1, and LDH (MFI) in YFP^+^ tumor cells (**B**) and (**C**–**E**) in tumor-infiltrating and spleen-derived CD8^+^, CD4^+^Foxp3^–^, and CD4^+^Foxp3^+^ T cells. For glucose-Cy3 staining for **C**, FOXP3–GFP C57BL/6J transgenic mice were used to identify Foxp3^+^CD4^+^ Tregs in live cells. (**F**) Representative flow cytometry histograms and quantified percentages of Ki67^+^ of B16-YFP^+^ cells and (**G**) representative flow cytometry histograms and quantified percentages of Ki67^+^ of tumor-infiltrating CD8^+^, CD4^+^Foxp3^–^, and CD4^+^Foxp3^+^ T cells from B16-YFP tumors implanted in mice (*n* = 5/group), as indicated in the schematic in **A**. (**H**) Representative flow cytometry contour plots of tumor-infiltrating CD8^+^ T cells stratified by high or low glucose-Cy3 uptake and (**I**) quantification of percentages of Cy3-high or -low out of total CD8^+^ T cells (*n* = 5). (**J**) Representative flow cytometry contour plots and quantified percentages of PD-1^+^ of tumor-infiltrating CD8^+^ T cells stratified by high or low glucose-Cy3 uptake. Data show a representative experiment of 3 independent experiments. All statistics produced by Wilcoxon’s rank-sum test implemented in GraphPad Prism. *P < 0.05; **P < 0.01; ****P < 0.0001. Data are represented as mean ± SEM.

We then asked how the adaptive immune compartment aids in slowing tumor growth upon LDH inhibition in vivo. We hypothesized that inhibiting LDH would slow tumor glucose uptake and therefore increase glucose availability as well as the ability of immune cells within the TME to consume glucose and better execute their tumoricidal functions. To investigate this, we compared glycolytic parameters among tumor cells and CD8^+^ T cells, CD4^+^Foxp3^–^ cells (effector T cells [Teffs]), and CD4^+^Foxp3^+^ cells (Tregs) within the TME from established B16-YFP tumors excised from mice treated with LDHi for 10 days (Figure 4A). When examining the YFP^+^ tumor cells from LDHi- versus vehicle control–treated tumors, we observed a decrease in ex vivo tumor glucose uptake based on glucose-Cy3 staining as well as a decrease in GLUT1 and LDH expression by flow cytometry (Figure 4B). Conversely, tumor-infiltrating T cell subsets from LDHi-treated mice displayed higher levels of glucose uptake compared with those from vehicle-treated mice, both ex vivo and in vivo (Figure 4C and Supplemental Figure 5A), as well as elevated GLUT1 expression (Figure 4D), while glucose uptake and GLUT1 expression in T cells from the spleen and whole blood remained unchanged (Figure 4, C and D, and Supplemental Figure 5B). In addition, while daily LDHi administration led to a decrease in tumor cell LDH expression, LDH expression in TILs as well as T cells from the spleen and whole blood remained unchanged (Figure 4E and Supplemental Figure 5D). This increase in T cell glucose uptake within LDHi-treated tumors was linked to an increase in glucose concentration in tumor interstitial fluid (Supplemental Figure 5C). Increases in glucose uptake and GLUT1 expression in TILs upon LDHi were confirmed in MC38 tumor–bearing mice as well, while CD45^–^ cells from the same tumors displayed decreased glucose uptake, GLUT1, and LDH (Supplemental Figure 5, E and F). We also found that in vivo daily treatment with LDHi versus vehicle increased the proliferative capacity of CD8^+^ and CD4^+^ Teff TILs while reducing tumor cell proliferation, as assessed by Ki67 expression (Figure 4, F and G). These data suggest that LDHi modifies tumor cell glycolysis and proliferation in vivo*,* such that tumor-infiltrating T cells are enabled to increase their glycolytic and proliferative capacity. We then asked whether increased glucose uptake was associated with increased levels of T cell activation. We divided tumor-infiltrating CD8^+^ T cells into 2 populations based on their levels of glucose-Cy3 uptake: Cy3-low versus Cy3-high (Figure 4H). As expected, we detected a higher percentage of Cy3-high tumor-infiltrating CD8^+^ T cells from mice treated with LDHi compared with vehicle treatment (Figure 4, H and I). We further observed that Cy3-high CD8^+^ T cells expressed higher levels of activation markers PD-1, CD44, CD25, and GITR independently of drug treatment, while maintaining similar levels of exhaustion markers Tim-3 and Lag-3 (Figure 4J and Supplemental Figure 5, G and H). Therefore, increasing glucose uptake capacity via LDH inhibition can polarize CD8^+^ T cells toward a more activated phenotype.

### LDH inhibition facilitates antitumor T cell functions.

**Figure 5:**
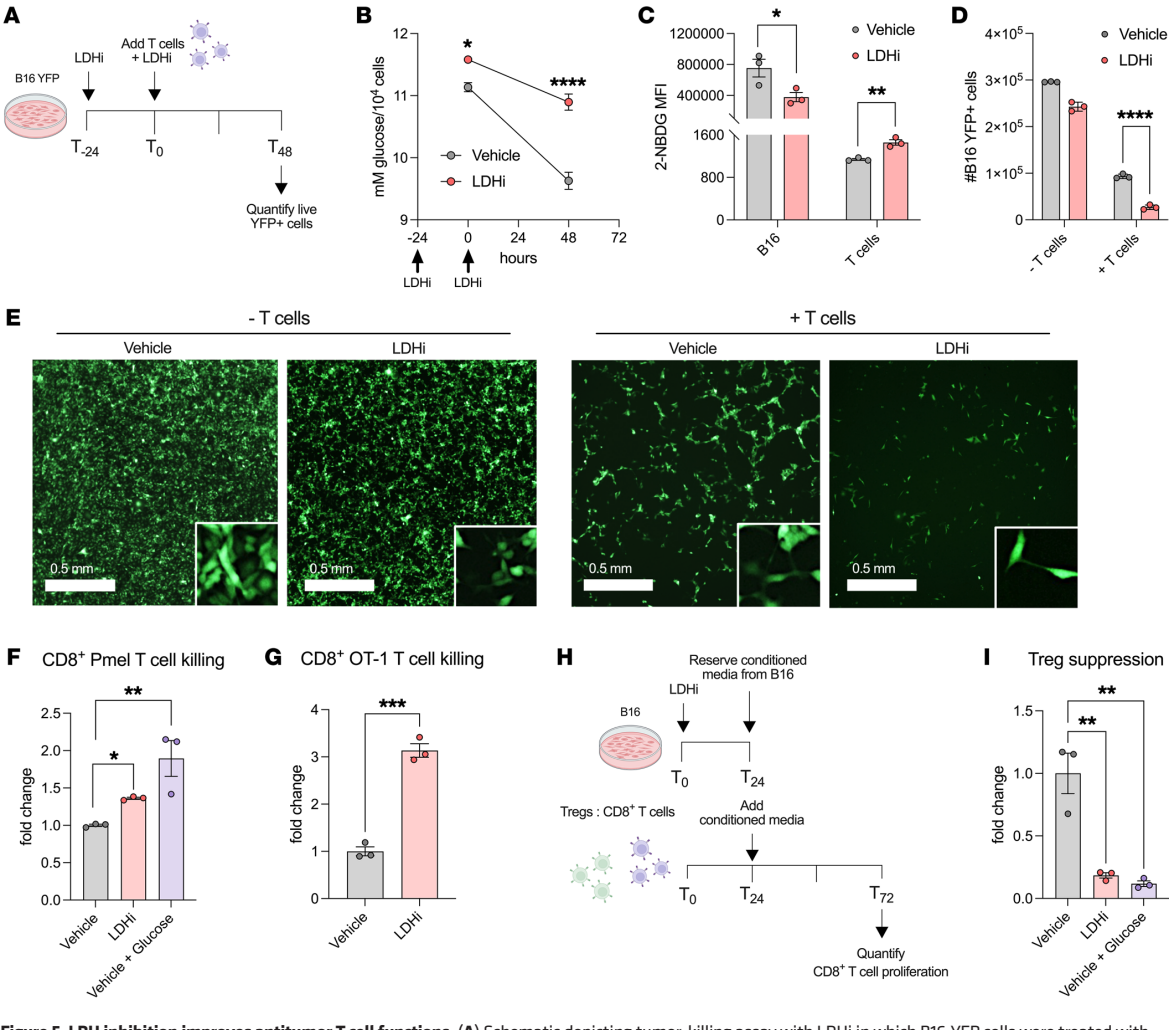
LDH inhibition improves antitumor T cell functions. (**A**) Schematic depicting tumor-killing assay with LDHi in which B16-YFP cells were treated with 20 μM LDHi or vehicle 24 hours apart and T cells were added 24 hours after the first LDHi treatment. (**B**) Quantified media glucose from killing assay coculture. (**C**) Flow cytometry quantification of 2-NBDG (MFI) in B16-YFP and CD8^+^ Pmel-1 T cells from killing assay cocultures 48 hours after last treatment. (**D**–**F**) (**D**) Quantified YFP^+^ tumor cells and (**E**) representative in vitro killing assay images of YFP^+^ tumor cells after 48 hours of coincubation with Pmel-1 CD8^+^ T cells as in **A**. (**F**) Corresponding quantified YFP^+^ tumor cells and percentages of tumor killing in the same conditions as above alongside vehicle supplemented with 10 mM glucose. (**G**) Quantification of killing of OVA_257-264_–pulsed live B16-YFP tumor cells by OVA-primed CD8^+^ T cells from OT1 transgenic mice upon 48 hours of coculture in the presence of LDHi (as indicated in **A**). E:T = 2:1, cocultured over 48 hours. (**H**) Schematic depicting in vitro Treg suppression assay with MACS column–sorted Tregs (CD4^+^CD25^+^ Regulatory T Cell Isolation Kit, mouse) cocultured with αCD3/αCD28-activated CTV-labeled syngeneic CD8^+^ T cells for 48 hours with the addition of conditioned media from B16 cells treated with 20 μM LDHi or vehicle or fresh media containing 10 mM glucose. (**I**) Percentage of suppression was calculated as percentage reduction in CD8^+^ T cell proliferation with respect to CD8^+^ T cells cultured alone in the same treatment and glucose conditions. Data show 1 representative experiment of 3 independent experiments (*n* = 3–4 technical replicates). All statistics produced by 2-way ANOVA with Bonferroni’s multiple-comparisons test implemented in GraphPad Prism. *P < 0.05; **P < 0.01; ***P < 0.001; ****P < 0.0001. Data are represented as mean ± SEM.

We then explored whether LDH inhibition and subsequent increased T cell glucose uptake would enhance the antitumor cytotoxic capacity of CD8^+^ T cells. Since we previously established that systemic LDHi treatment leads to increased tumor-infiltrating T cell glucose uptake and proliferation, we determined whether increasing glucose levels (by either LDHi treatment or glucose supplementation) in culture would facilitate T cell cytotoxicity in vitro. For this, we established a tumor antigen–specific CD8^+^ T cell killing assay, using antigen-primed melanoma-specific Pmel-1 TCR transgenic T cells and target B16 cells as previously described (46). We found that 24 hours of B16 pretreatment with LDHi followed by another LDHi treatment in B16:T cell cocultures maintained higher glucose levels compared with vehicle over 48 hours of coculture (Figure 5, A and B). This was linked to a significant decrease in tumor cell glucose uptake as evaluated by fluorescent glucose tracker 2-NBDG (Figure 5C). Conversely, we observed an increase in 2-NBDG glucose uptake by the CD8^+^ Pmel-1 T cells in the same conditions, mirroring the effects of LDHi treatment in vivo (Figure 5C). In addition to facilitating an increase in glucose availability and T cell glucose uptake, administration of LDHi to the coculture led to an increase in antitumor T cell killing (Figure 5, D–F), with no difference in T cell viability observed between the LDHi and vehicle treatment conditions (Supplemental Figure 6A). We also confirmed an increase in T cell killing when additional glucose was added to the cocultures instead of LDHi (Figure 5F). This increase in T cell killing upon LDHi treatment was confirmed with another TCR transgenic model using ovalbumin-specific CD8^+^ T cells cultured with B16 that had been pulsed with the corresponding OVA-peptide (SIINFEKL) prior to the coculture (Figure 5G and Supplemental Figure 6B).

Since intratumoral CD8^+^ T cells and Tregs both increase their glucose-uptake capacity upon LDHi treatment (Figure 4C), we looked to see how increased glucose availability upon LDHi might directly affect Treg function as well. Despite increasing the proliferation capacity of other TILs, LDH inhibition did not increase Treg proliferation in vivo (Figure 4G). Previous work has demonstrated that Treg functional stability (25) and suppressive capacity (40) are both reduced when Tregs engage in glucose catabolism. In order to mechanistically evaluate the functional effects of LDHi treatment on Tregs in the TME, we performed a Treg suppression assay with conditioned media from tumor cells treated with LDHi to mimic the in vivo microenvironment (Figure 5H). Addition of the conditioned media from B16 tumor cells treated with LDHi versus vehicle for 24 hours to the Treg: CD8^+^ T cell coculture abrogated Treg-mediated suppression of CD8^+^ T cell proliferation (Figure 5I and Supplemental Figure 6C). Similarly, supplementing 10 mM glucose-containing media to the vehicle-treated tumor cell conditioned media abrogated Treg suppression as well (Figure 5I and Supplemental Figure 6C). These data suggest increasing intratumoral glucose levels using LDHi is not only more advantageous for CD8^+^ T cell–mediated killing, but also detrimental for Treg-suppressive function.

### LDHi improves responses to ICB.

**Figure 6:**
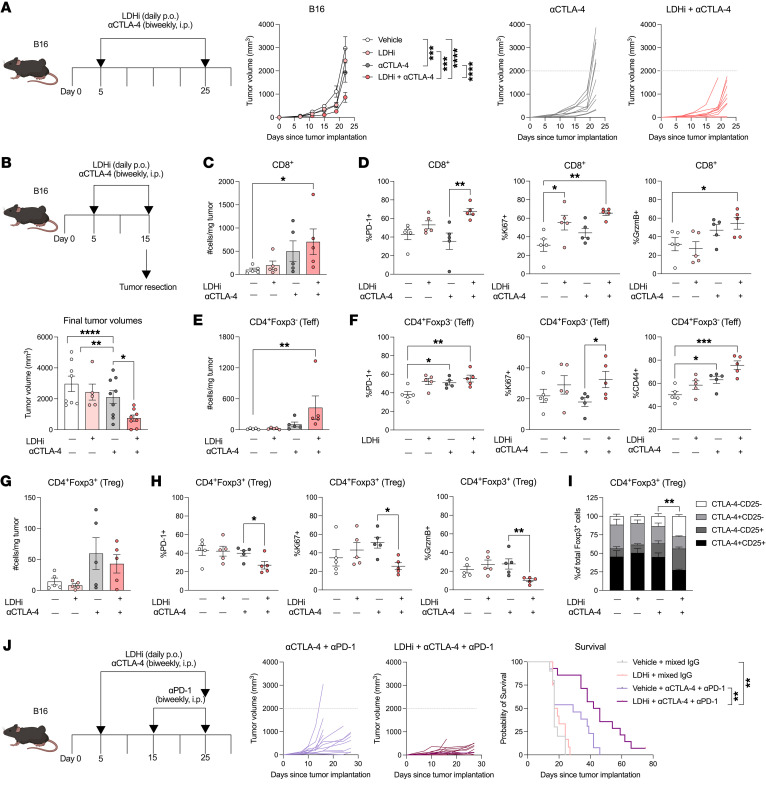
LDHi improves the therapeutic and immune activity of ICB. (**A**) Averaged and individual tumor growth curves (mm^3^) from B16 tumor-bearing mice treated with 100 mg/kg LDHi and/or CTLA-4 blockade (9D9, IgG2b) or control vehicle/IgG as indicated in the schematics (*n* = 10 mice/group). (**B**–**I**) Treatment schematic for TME analyses and B16 tumor volume at the end of treatment (**B**). (**C**–**I**) Flow cytometry quantification of CD8^+^, CD4^+^Foxp3^–^, and CD4^+^Foxp3^+^ T cell absolute numbers and their expression of PD-1, Ki67, granzyme B, CD44, CTLA-4, and/or CD25 from B16-treated tumors as in **B** (*n* = 5 mice/group). Data show 1 representative experiment of 3 independent experiments. (**J**) Tumor growth and survival curves from B16-bearing mice treated with LDHi (100 mg/kg) or vehicle with or without αCTLA-4 (100 μg, clone 9D9) + αPD-1 (250 μg, clone RMP1-14), or respective IgG controls as indicated in the schematic (*n* = 10–15 mice/group). Data show 1 representative experiment of 2 independent experiments. All statistics produced by 2-way ANOVA with Bonferroni’s multiple-comparisons test implemented using GraphPad. *P < 0.05; **P < 0.01; ***P < 0.001; ****P < 0.0001. Data are represented as mean ± SEM.

Finally, we investigated whether LDH inhibition could improve the antitumor activity of ICB in solid tumor models that are typically resistant to these therapies. Previous work has indicated that tumor glycolysis confers resistance to CTLA-4 blockade, while tumor cell oxidative metabolism is a mechanism of resistance to αPD-1 therapy (25, 38). In line with this rationale, we investigated the approach of combining LDHi with CTLA-4 blockade. According to our hypothesis, we found that LDHi enhances the activity of CTLA-4 blockade in delaying B16 and MC38 tumor growth, with greater antitumor effects of the combination than each treatment alone (Figure 6A and Supplemental Figure 7A). Upon examining the tumor-immune infiltrate from B16 tumors after 10 days of treatments, we observed an increase in CD8^+^ and CD4^+^ Teffs in the combination treatment group that was not detectable in either monotherapy treatment group (Figure 6, B–E). We also observed PD-1 and Ki67 upregulation in CD8^+^ and CD4^+^ Teffs from the combination-treated tumors (Figure 6, D–F). Additionally, CD4^+^ Teffs also upregulated CD44 expression, while CD8^+^ T cells upregulated granzyme B upon use of the combination treatment (Figure 6, D–F).

Interestingly, Tregs deviated from these trends. While their infiltration was only mildly reduced between αCTLA-4 alone and the combination treatment (Figure 6G), LDHi combined with CTLA-4 blockade led to pronounced and consistent decreases in Treg activation (PD-1), proliferation (Ki67), and granzyme B expression and was associated with predominant loss of Tregs expressing suppressive markers CD25 and/or CTLA-4 (Figure 6, H and I). Taken together, these data indicate that LDH inhibition can potentiate the antitumor activity of CTLA-4 blockade by increasing Teff activation while destabilizing the Treg phenotype.

While inhibiting LDH did improve responses to CTLA-4 blockade, we found that combining LDHi with αPD-1 did not lead to an enhanced delay of B16 tumor growth (Supplemental Figure 7B). Since LDHi with CTLA-4 blockade drove an increase in PD-1 expression on CD8^+^ and CD4^+^ Teffs, we tested to determine whether adding PD-1 blockade to this combination would further enhance the antitumor activity. We observed that incorporating PD-1 blockade starting from the time point in which we observed PD-1 upregulation in T cells upon LDHi and aCTLA-4 significantly delayed tumor growth and prolonged survival compared with ICB alone (Figure 6J). Overall, these data support the use of LDHi to combat resistance to the clinically relevant combination of αPD-1 and αCTLA-4.

## Discussion

Our results highlight the potential of LDHA as a rational target for combination with immunotherapy to improve antitumor immunity. Altered tumor metabolism is a major hallmark of cancer, and tumor cells rely on glycolytic enzymes, such as LDH, to support cancer progression (4, 6, 31). However, it was unclear whether targeting this pathway systemically could enhance the antitumor immune response, given that this approach can affect both tumor cells and immune cells. Since T cells engage in glycolysis to perform their effector functions (35–37), there remained a critical need to determine how to preferentially target tumor glycolysis while avoiding inhibition of T cell glycolysis and function. We demonstrate that pharmacologically targeting LDHA redirects intratumoral glucose utilization from tumor cells to T cells, favoring antitumor immunity. This highlights the promising strategy of combining LDHi with ICB for treating highly glycolytic cancers in patients.

We observed that cancer cells overexpress LDHA compared with nonmalignant cells within the tumor, indicating its preferential utilization by tumor cells for glycolysis. We also demonstrated functional overreliance on glycolysis by tumor cells compared with T cells, both in vitro using human and mouse cells and ex vivo from murine syngeneic tumor models, identifying a therapeutic window for preferentially targeting LDHA in tumor cells. It is well known that cancer patients with elevated serum LDH have a poorer prognosis and are less likely to respond to immunotherapies (3, 12, 27–30, 33, 47). Here, we show that there exists a direct correlation between serum and tumor LDH in a mouse melanoma model and that, in melanoma patients, tumor LDHA is associated with shorter survival. We also found that glycolysis genes negatively correlate with genes related to immune cell infiltration and activation in melanoma patient samples, indicating that tumor glycolysis may hinder immune responses, supporting the targeting of this pathway to enhance antitumor immunity. Since the function and enzymatic activity of tumor LDH directly contribute to cancer progression and immune suppression (7, 14, 31, 20, 48), we hypothesized that inhibiting LDH would both destabilize tumor progression and reverse suppression of immune cells.

However, despite tumor overreliance on glycolysis and LDH specifically, LDHis remain absent from clinical trials for a variety of reasons, including potential off-target toxicity and the high doses needed to achieve therapeutic efficacy in mice (45, 49). Before investigating LDHi as a potential combinatorial agent with immunotherapy, we wanted to understand the isolated effects of LDHi on T cells, particularly on T cell glycolysis. Several studies have reported T cell reliance on glycolysis, particularly upon activation (37, 50). However, T cells may be less sensitive to metabolic perturbations and thus may respond differently than tumor cells to metabolic interventions. Previous work has shown that antimetabolic therapies, such as inhibitors of oxygen consumption or glutamine metabolism, can enhance T cell function while impairing tumor cells (51, 52). Similarly, we demonstrate a differential response to LDH inhibition between tumor cells and immune cells. Tumor cells display higher glycolytic sensitivity to LDH inhibition, resulting in decreased glucose uptake and glycolytic flux, while T cells showed relative resistance to the same treatment. These data provide further rationale for LDHi as potential therapeutic agents to combine with immunotherapy.

Several groups previously demonstrated that LDHis have an antitumor effect (16, 34, 44, 45), but the contribution of the adaptive immune system to this mechanism, if any, was not described. Here, we find that an LDHi delays tumor growth in preclinical models of melanoma and colon cancer. In B16 melanoma tumors, this effect was dependent on both tumor LDHA expression and the presence of the adaptive immune compartment.

We show that in vivo, pharmacologic LDH inhibition using GNE-140 leads to decreased glucose uptake, GLUT1, and LDH expression in tumor cells while simultaneously increasing glucose uptake and GLUT1 expression in tumor-infiltrating T cells due to increased glucose availability in the TME. These findings suggest that LDH inhibition preferentially targets tumor cells over immune cells within the TME and specifically changes tumor cell metabolism while preserving or even enhancing intratumoral T cell metabolism.

In addition to elevated serum LDH, increased glucose uptake is also an established biomarker for cancer (5, 6, 53). FDG-PET imaging techniques are widely employed in the clinic to identify areas where cancer may be progressing (54). However, inhibitors of glucose uptake have not proven to be efficacious in the clinic due to off-target toxicity (20). This may be due to the fact that all cells uptake glucose to produce energy, but the fate of glucose may differ between oncogenic and healthy cells. Therefore, we propose LDH inhibition as an alternative method for blunting glucose consumption by tumor cells, while sparing immune cells due to their relatively lower expression of LDH. Glucose is a limiting nutrient for CD8^+^ T cell and CD4^+^ Teff activation and cytotoxicity (55), while Tregs thrive under low-glucose conditions (25, 41). In B16 melanoma and MC38 colon cancer, LDH inhibition resulted in increased glucose-uptake capacity in all 3 T cell subtypes, suggesting potentiation of antitumor Teff function and reduction of Treg-mediated immunosuppression. Our results further emphasize the importance of intratumoral glucose availability in modulating T cell responses. Accordingly, we show that while increasing media glucose levels enhances CD8^+^ T cell–mediated killing of tumor cells, Treg-mediated suppression is less effective in the same setting. We also demonstrate that administration of LDHi in a competitive tumor: T cell coculture setting is an effective strategy for increasing glucose availability and thereby increases CD8^+^ T cell killing, while also reducing Treg-mediated suppression of CD8^+^ T cells. These findings suggest that interventions aimed at increasing intratumoral glucose levels or switching its utilization from tumor to immune cells may improve the efficacy of immunotherapies, including settings where T cells are adoptively transferred into patients.

Based on our prior findings showing that CTLA-4 blockade is more effective against LDHA-KD tumors in immunocompetent mice (25), here we tested to determine whether we could achieve similar results by combining αCTLA-4 therapy with pharmacologic LDH inhibition. Aligning with our hypothesis, we found that combining CTLA-4 blockade with LDHi delays tumor progression in B16 melanoma and MC38 colon cancer more effectively than using each agent alone. This combination therapy led to increased infiltration and activation of CD8^+^ T cells and Teffs within the tumor, indicating enhanced antitumor immune responses. Additionally, the combination therapy altered Treg-suppressive phenotypes, indicating possible reduction of their suppressive function. This is in accordance with our previous findings related to Treg dysfunction in glycolysis-low tumors. Our group and others have demonstrated a glucose-dependent decrease in Treg suppression in vitro (25, 40), which is further supported by our data. These results suggest that LDH inhibition in combination with CTLA-4 blockade promotes Teff activation while impairing Treg function, which is crucial in overcoming the immunosuppressive TME. Moreover, adding PD-1 blockade to the αCTLA-4 and LDHi combination regimen further delays tumor growth and leads to improved survival in mice bearing melanoma tumors, indicating that priming the TME with αCTLA-4 and LDHi may enhance antitumor responses to PD-1 blockade.

In conclusion, our study highlights LDH inhibition as a rational strategy for preferential targeting of the overactive glycolytic pathway in cancer cells and improving antitumor immunity. Our results demonstrate that LDH inhibition can effectively limit tumor progression and serves as a tumor-specific strategy for redirecting glucose uptake from cancer cells to immune cells within the TME. Additionally, the differential glycolytic sensitivity between tumor and T cells supports further clinical development of LDHis in combination with immunotherapy. Overall, these findings offer a promising direction for future research in developing novel therapeutic approaches for highly glycolytic cancers and improving the outcomes of immunotherapy in cancer patients.

## Methods

### Sex as a biological variable.

Our study examined male and female humans and mice, and similar findings are reported for both sexes.

### Bulk RNA-Seq analysis.

To compare gene expression across normal and malignant tissues, TPM count matrices and annotation data were obtained from GTEx (Analysis V8) and CCLE (DepMap Public 22Q4), respectively. Both data sets were log_2_ normalized to ensure comparability, and each sample was labeled using the annotation data. The normal tissue samples were grouped into tissue types based on the Tissue Site Detail field (SMTSD), whereas cancer cell lines were grouped into cancer types based on Cellosaurus NCIt disease field (Cellosaurus_NCIt_disease). For correlative comparison of immune characteristics, mRNA expression levels and survival data were collected from cBioportal for skin cutaneous melanoma SKCM (TCGA, Provisional) (*n* = 472). Correlation plots to consider expression of immune and glycolytic gene markers were generated using the R package ggcorplot. For survival analysis in the context of gene expression, TPM count matrices and annotation data were obtained from Riaz et al. (56). Samples were filtered for those “on treatment” and defined as LDHhi or LDHlo by expression of LDHA above or below the median LDHA value across all samples, respectively. Survival plots and log-rank statistical test were generated using survfit and ggsurvplot functions from the survival and survminer packages in R. Visualizations were generated using the R statistical platform and GraphPad Prism 10.

### scRNA-Seq analysis.

To compare gene expression across the TME, the scRNA-Seq counts matrix and reported cell phenotypes were imported from Jerby-Arnon et al. (57). To identify a Treg subpopulation, we created subclusters of cells using the Louvain algorithm (58) at default resolution, and a subcluster of T.CD4 cells was identified based on unique CD4 and FOXP3 coexpression. Downstream analysis and visualization were performed on the data set using R package Seurat, version 4.3.0 (https://github.com/satijalab/seurat/tree/release/4.3.0; commit ID ff03fdf21f1b8fea9ee247d0fd83df5811507027).

### Cell lines and reagents.

The B16F10 mouse melanoma line was originally obtained from I. Fidler (Department of Cell Biology at the University of Texas MD Anderson Cancer Center, Houston, Texas, USA). B16 cells expressing YFP (B16-YFP) were generated as previously described (59). B16F10 cells were transfected with SureSilencing LDHA-targeting shRNA plasmids (KD; A2 = GTACGTCCATGATGCATATCT; A3 = TGCCAACTGCAGGCTTCGATT) or scramble control plasmids (Sc = GGAATCTCATTCGATGCATAC) (QIAGEN). Stable LDHA-KD (B16-KD) and scramble control (B16-Sc) cell lines were generated as previously described (9, 26). The colon cancer cell line MC38 was obtained from the National Cancer Institute (NCI) (Bethesda, Maryland, USA). Cells were cultured in RPMI-1640 medium containing 7.5% FCS supplemented with 2 mM l-glutamine and penicillin/streptomycin. Cell lines were routinely mycoplasma tested. Cells were detached using 0.25% trypsin/EDTA. For cell surface staining and killing assays, cells were detached nonenzymatically using Cellstripper. LDHi GNE-140 (S6675) was obtained from Selleckchem and dissolved in DMSO for use according to the manufacturer’s instructions. LDHA modulation in B16-KD– or LDHi–treated cells was confirmed at the protein level by Western blot using a rabbit anti-LDHA antibody (1:1,000; Cell Signaling Technology, 2012S) coupled with HRP-conjugated anti-rabbit IgG (1:5,000; Cell Signaling Technology, 7074S) as a secondary antibody, with vinculin (1:1,000, Santa Cruz Biotechnology Inc., sc-73614; revealed by an HRP-conjugated anti-mouse IgG, 1:5,000, Cell Signaling Technology, 7076S) or β-actin (1:5,000, MilliporeSigma, A2103; revealed by Molecular Devices Evaluation Kit R8202) as a protein loading control, and at the enzymatic activity level by using the Cytotoxicity Detection Kit PLUS (LDH) (Roche Diagnostics), as previously reported (26). Altered glycolytic and mitochondrial metabolism capacity of these tumor cells was also confirmed by Glycolytic Rate Assay, Glycolysis Stress Test, Mitochondrial Stress Test, and ATP rate assays using a Seahorse XF 96 Analyzer according to the manufacturer’s instructions (Agilent Technologies).

### Mice.

C57BL/6J mice were purchased from The Jackson Laboratory. FOXP3-GFP transgenic mice were provided by A. Rudensky and backcrossed to C57BL/6J at Memorial Sloan Kettering Cancer Center (MSKCC). Same-sex, same-aged mice were used in each experiment. All mice were bred and maintained under specific pathogen–free conditions (with a 12-hour light/12-hour dark cycle at temperature of 21°C–23°C and humidity of 35%–55%) and used at the ages of 5–10 weeks. The maximal tumor size of 20 mm in any direction was not exceeded in any experiment.

### In vivo experiments.

For syngeneic tumor experiments, 8– to 10-week-old female C57BL/6 (JAX, 00664) mice and Rag1^−/−^ (JAX, 002216) mice were intradermally (i.d.) implanted with 0.5 × 10^6^ B16 or MC38 cells on the right flank. Tumor diameter was measured by calipers twice per week, and tumor volume was calculated using volume = (*L* × *W^2^*)/2, where *L* is tumor length and *W* is tumor width. Mice were randomized in the different treatment groups to receive 3 i.p. injections with 100 μg anti–CTLA-4 (clone 9D9 IgG2b, Bio X Cell) or isotype control (clone MPC-11, Bio X Cell) and/or 250 μg anti–PD-1 (clone RMP1-14 IgG2b, Bio X Cell) or isotype control (clone 2A3, Bio X Cell) twice a week. LDHi ([R]-GNE-140) was administered at 100 mg/kg in 0.5% methylcellulose and sterile water by oral gavage (p.o.) daily for the duration of the experiments as indicated. Both antibody and small molecule treatments began on day 5 after tumor implantation.

### Metabolite quantification.

Glucose and lactate were quantified in culture supernatants by either luminescent assays (Glucose-Glo, Promega) or by YSI meter (MSKCC Metabolism Core Facility). The Glucose-Glo Assay (Promega) was used to quantify glucose consumption in supernatants from B16-Sc and B16-KD cells. Glucose consumption was calculated with the following formula: glucose consumptio*n* = (glucose in base medium − glucose in conditioned medium)/no. of cells. YSI-based measurements of glucose consumption and lactate production were calculated as follows: glucose consumptio*n* = (glucose in conditioned medium − glucose in base medium)/(no. of cells/106 × hours). Lactate productio*n* = (lactate in conditioned medium − lactate in base medium)/(no. of cells/106 × hours). Intracellular metabolites were quantified by mass spectrometry (MSKCC Metabolism Core Facility).

### Flow cytometry analyses.

For flow cytometry analysis of in vitro assays described above, tumor cells were detached with Cellstripper (Corning) or T cells were collected, and both cell types were stained with Zombie NIR viability dye for 15 minutes in PBS, washed, and stained with fluorophore-conjugated surface antibodies for 30 minutes on ice in FACS buffer (PBS + 0.5% BSA + 2 mM EDTA). For flow cytometry analysis of drug-treated tumor-bearing mice, spleens and tumors were isolated. Tumors were weighed, and single-cell suspensions were prepared by mechanical dissociation through 40 μm filters for spleens and 100 μm filters for tumors. Tumors were further purified using 40% Percoll gradient centrifugation at 2,000*g*. Red blood cells were removed from spleens using ACK lysis buffer. Cells were plated and pelleted in 96-well V-bottom plates and stained with Zombie NIR Viability Dye (BioLegend) for 15 minutes in PBS on ice, then washed with FACS buffer. Cells were then blocked in 5 mg/ml Fc-block antibody (2.4G2, MSKCC Antibody Core Facility) for 15 minutes on ice in FACS buffer. Cells were then stained with half of the surface antibodies in FACS buffer for 30 minutes on ice, washed, stained with the other half of surface antibodies in FACS buffer, and washed 2× with 200 μL FACS buffer. All intracellular staining was conducted using the Foxp3 Fixation/Permeabilization Staining Buffer Set (eBioscience, 00-5523-00) according to the manufacturer’s protocol. The blocking buffer from the block step was supplemented with 1% mouse serum (Thermo Scientific, 24-5544), 1% rat serum (Thermo Scientific, 24-5555), 1% human serum (Thermo Scientific, BP2525100), and 100 U/mL heparin (MilliporeSigma, H3393). All flow antibodies used are listed in Supplemental Table 1. Flow cytometry was performed on a BD LSRII or Cytek Aurora. Glucose analog staining was performed before viability or surface staining as follows: 2-NBDG staining was performed by incubation with 100 μM 2-NBDG (Invitrogen) in complete RPMI in a humidified incubator at 37°C for 15 minutes or injected via tail vein (500 nmol per injection) to quantify glucose uptake in vivo. Glucose-Cy3 staining was performed by 25 minutes of incubation in serum-free, glucose-free RPMI 1640 containing 0.4 μM glucose-Cy3 (provided by G. Delgoffe, University of Pittsburgh Medical Center, Hillman Cancer Center, Pittsburgh, Pennsylvania, USA) in a humidified incubator at 37°C. For both assays, cells were then extensively washed before acquisition. All analyses were completed using FlowJo software, version 10.

### Killing assays.

Single-cell suspensions of splenocytes were isolated from OT-1 TCR transgenic mice purchased from The Jackson Laboratory or Pmel-1 TCR transgenic mice obtained from N. Restifo (NIH, Bethesda, Maryland, USA). Splenocytes were primed with OVA (SIINFEKL 257-264, AnaSpec, AS-60193-1) or human gp100 peptide (25-33, AnaSpec, AS-62589) in RPMI media supplemented with 10% FCS and 50 mM BME as previously described (46). B16-YFP target cells were detached with cell stripper, plated at 5,000 cells per well in a 24-well plate, and pretreated with LDHi for 24 hours. For OT-1 cultures, tumor cells were pulsed with 0.01 μg/mL OVA for 2 hours at 37°C in media. B16-YFP cells were quantified on a Celigo Image Cytometer (Nexcelom) at the following time points: 0 hours, 24 hours, 48 hours, and 72 hours. At time-point 0 hours, OT-1 T cells were added at a final effector/target ratio (E:T) of 2:1; Pmel T cells were added at a final E:T of 5:1. Percentage of target cell killing was calculated and normalized to B16 growth without the addition of T cells.

### In vitro T cell assays.

Suppression assays were performed by incubating Tregs at a 1:1 ratio with immunomagnetically purified CD45.1^+^CD8^+^ T cells (CD8 microbeads, Miltenyi Biotec) or CD45.1^+^CD4^+^ T cells (CD4 microbeads, Miltenyi Biotec), which were labeled with CellTrace Violet (CTV) (Invitrogen) as previously reported (60). Cultures were stimulated with αCD3/αCD28 microbeads (Dynabeads Mouse T-Expander CD3/CD28, Thermo Fisher, 11456D) in complete RPMI 1640 containing the indicated concentrations of glucose (MilliporeSigma) for 48 hours in a humidified chamber with 5% CO_2_ at 37°C. After incubation, cultures were processed for flow cytometry analyses of CTV dilution of CD8^+^ T cells (CD8^+^ T cell proliferation). Treg suppression was calculated with the following formula: %Treg suppression = (1 − %CTV_low_[CD8^+^ T cells + Tregs]/%CTV_low_[CD8^+^ T cells alone]) × 100

### Statistics.

Statistical significance was determined using unpaired *t* tests with Welch’s correction, Wilcoxon’s rank-sum test, or 2-way ANOVA, as indicated in the figure legends. Statistical analyses were performed using Prism 10 software (GraphPad Software), version for Macintosh Pro. Detailed information for statistical tests and numbers of observations or replicates used in each experiment and definition of center and dispersion are reported in the figure legends. *P* < 0.05 was considered significant.

### Study approval.

Human melanoma RNA-Seq data sets investigated in this study were previously reported (42). Mice were housed under specific pathogen–free conditions in the animal facility of MSKCC, and all animal experiments were conducted according to protocols approved by the MSKCC and Weill Cornell Medicine Institutional Animal Care and Use Committee. (MSKCC protocol 2022-0025).

### Data availability.

Values for all data points in graphs are reported in the Supporting Data Values file. All represented data are available upon request.

## Author contributions

SV, RZ, JDW, and TM conceptualized the study. SV, RZ, I Schulze, I Serganova, SB, and LD provided methodology. SV performed the experiments and analyzed the data. LMM, LD, JFK, MAB, AA, and YM assisted with conducting experiments and select analyses. JDW and TM acquired funding and supervised the study. SV wrote the original draft of the manuscript. JDW, TM, and RZ reviewed and edited the manuscript.

## Supplementary Material

Supplemental data

Unedited blot and gel images

Supporting data values

## Version 1 09/03/2024

Electronic publication
