# Supplementary material for: Pharmacologic LDH inhibition redirects intratumoral glucose uptake and improves antitumor immunity in solid tumor models
Source: J Clin Invest. 2024 Sep 3;134(17):e177606. doi: 10.1172/JCI177606 (PMC11364391; doi:10.1172/JCI177606)
Supplement: Supplemental data [file jci-134-177606-s244.pdf]

## Supplemental Material

**Full conflict-of-interest statement:** RZ is an inventor on the following patents and patent applications: “Treatment Of Glycolysis-Low And Glycolysis-High Tumors With CTLA-4 Inhibitors” (US provisional application 63/310,089); “CD4+ TFH-Like Cells as a Therapeutic Target (international application PCT/US2021/055992); “Inhibition of CTLA-4 and/or PD-1 for Regulation of T cells” (US provisional patent 62/582,416); “Antibodies and Methods of Use Thereof” (US provisional patent application WO2018106864A1); “Anti-PD-1 Antibodies and Methods of Use Thereof” (US patent application US10323091B2); and “Anti-GITR Antibodies and Methods of Use Thereof” (patent application US20180244793A1). RZ is a scientific advisory board member of iTEOS Therapeutics and receives grant support from AstraZeneca and Bristol Myers Squibb. JDW is a consultant for Apricity; Ascentage Pharma; AstraZeneca; BeiGene; Bicara Therapeutics; Bristol Myers Squibb; Chugai, Daiichi Sankyo; Dragonfly; Imvaq; Larkspur; Psioxus, Recepta; Tizona; Trishula Therapeutics; and Sellas. JDW received grant/research support from Bristol Myers Squibb. JDW has equity in Apricity, Arsenal IO; Ascentage; CellCarta; Imvaq; Linneaus, Larkspur; Georgiamune; Maverick; Tizona Therapeutics; and Xenimmune. JDW is an inventor on the following patents: “Xenogeneic DNA Vaccines,” “Newcastle Disease Viruses for Cancer Therapy,” “Myeloid-Derived Suppressor Cell (MDSC) Assay,” “Prediction Of Responsiveness to Treatment With Immunomodulatory Therapeutics and Method of Monitoring Abscopal Effects During Such Treatment,” “Anti-PD1 Antibody”; “Anti-CTLA4 Antibodies,” and “Anti-GITR Antibodies and Methods of Use Thereof”. TM is a consultant for Immunos Therapeutics, Daiichi Sankyo Co., TigaTx, Normunity, and Pfizer; is a cofounder of and equity holder in IMVAQ Therapeutics; receives research funding from Bristol Myers Squibb, Surface Oncology, Kyn Therapeutics, Infinity Pharmaceuticals, Peregrine Pharmaceuticals, Adaptive Biotechnologies, Leap Therapeutics, and Aprea Therapeutics; and is a named inventor on patents listed in the table below.

# Taha Merghoub Patents

https://patents.google.com/?inventor=Taha+Merghoub&oq=Taha+Merghoub+

| Title: | Application number: | Filing Entity: |
|---|---|---|
| Antibodies and methods of use thereof | US11447557B2 | Agenus Inc. |
| Replication competent attenuated vaccinia viruses with deletion of thymidine kinase with and without the expression of human Flt3L or GM-CSF for cancer immunotherapy | US11986503B2 | Memorial Sloan Kettering Cancer Center |
| Anti-CTLA-4 antibodies and methods of use thereof | US11267889B2 | Agenus Inc. |
| Methods of administering anti-OX40 antibodies | US11472883B2 | Agenus Inc. |
| Anti-CTLA-4 antibodies and methods of use thereof | US11638755B2 | Agenus Inc. |
| Anti-OX40 antibodies and anti-GITR antibodies | US11359028B2 | Agenus Inc. |
| Anti-OX40 antibodies and methods of use thereof | US20210324097A1 | Agenus Inc. |

| | | |
|---|---|---|
| Anti-pd-1 antibodies and methods of use thereof | US20220372149A1 | Agenus Inc. |
| Recombinant MVA or MVADELE3L expressing human FLT3L and use thereof as immuno-therapeutic agents against solid tumors | US12036279B2 | Memorial Sloan Kettering Cancer Center |
| Neoantigens and uses thereof for treating cancer | EP3576781B1 | Icahn School of Medicine at Mount Sinai |
| Use of inactivated nonreplicating modified vaccinia virus Ankara (MVA) as monoimmunotherapy or in combination with immune checkpoint blocking agents for solid tumors | US11426460B2 | Memorial Sloan Kettering Cancer Center |
| Antibodies and method of use thereof | US20200024350A1 | Agenus Inc. |
| Use of MVA or MVAΔE3L as immunotherapeutic agents against solid tumors | US11253560B2 | Memorial Sloan Kettering Cancer Center |
| Anti-GITR antibodies and methods of use thereof | AU2022215304A1 | Agenus Inc. |
| Anti-GITR antibodies and methods of use thereof | US20200123265A1 | Agenus Inc. |
| Methods and compositions for tumor therapy | AU2016304597B2 | Memorial Sloan Kettering Cancer Center |
| Anti-OX40 antibodies and method of use thereof | EP3383914A1 | Agenus Inc. |

| | | |
|---|---|---|
| Anti-GITR antibodies and methods of use thereof | WO2017096276A1 | Agenus Inc. |
| Recombinant poxviruses for cancer immunotherapy | US20220056475A1 | Memorial Sloan Kettering Cancer Center |
| BI-Specific activators for tumor therapy | US20240239910A1 | Memorial Sloan Kettering Cancer Center |
| Alphavirus Replicon Particles Expressing TRP2 | US20200113984A1 | Alphavax, Inc. |
| Oncolytic Vaccinia virus expressing immune checkpoint blockade for cancer Immunotherapy | EP3765047A1 | Memorial Sloan Kettering Cancer Center |
| Inducing favorable effects of tumor microenvironment via administration of nanoparticle compositions | EP3765047A1 | Memorial Sloan Kettering Cancer Center |
| Vaccinia Virus mutants useful for cancer immunotherapy | US11884939B2 | Memorial Sloan Kettering Cancer Center |
| Methods and compositions for treatment of pancreatic cancer | IL289251A | Memorial Sloan Kettering Cancer Center |
| Inhibition of CTLA-4 and/or PD-1 for Regulations of T Cells | US20210179714A1 | Memorial Sloan Kettering Cancer Center |
| Heat-inactivated vaccinia virus as a vaccine immune adjuvant | EP3706768A1 | Memorial Sloan Kettering Cancer Center |
| CD40 binding molecules and uses of thereof | CA3202384A1 | Memorial Sloan Kettering Cancer Center |
| Antigen-binding proteins targeting melanoma differentiation antigens and uses thereof | US20190375855A1 | Memorial Sloan Kettering Cancer Center |

| | | |
|---|---|---|
| CD40 binding molecules and uses thereof | AU2021400733A9 | Memorial Sloan Kettering Cancer Center |
| Anti-PD-1 antibodies and methods of use thereof | AU2021200281B2 | Agenus Inc. |
| Anti-CTLA-4 antibodies and methods of use thereof | NZ794974A | Agenus Inc. |
| Peripheral blood phenotype linked to outcomes after immunotherapy treatment | US20240085417A1 | Memorial Sloan Kettering Cancer Center |
| CD4+ TFH- like cells as a therapeutic target | US20230390390A1 | Memorial Sloan Kettering Cancer Center |
| Antigen recognizing receptors targeting b7-h3 and uses there of | WO20224102685A2 | Memorial Sloan Kettering Cancer Center |
| Anti-PD-1 Antibodies and their methods of use | AR105886A1 | Agenus Inc. |
| Treatment of glycolysis-low and glycolysis-high tumors with CTLA-4 Inhibitors | WO2023154959A2 | Memorial Sloan Kettering Cancer Center Office of Technology Development |
| Il33 Proteints and methods of use thereof | WO2023081802A2 | Memorial Sloan Kettering Cancer Center |
| Models for predicting mutant p53 fitness and their implications in cancer therapy | WO2022177989A1 | Memorial Sloan Kettering Cancer Center |
| Chimeric receptor targeting muc16 and uses thereof | US20240189426A1 | Memorial Sloan Kettering Cancer Center |
| Heteroclitic cancer vaccines | EP4121081A1 | Memorial Sloan Kettering Cancer Center |

| | | |
|---|---|---|
| Use of Composition, MVA?E3L and Pharmaceutically acceptable Vehicle of Diluent | BR112017022134B1 | Memorial Sloan Kettering Cancer Center |
| Antibodies and Methods of its use | AR110348A1 | Agenus Inc. |
| Phosphatidylserine targeting agents and uses thereof for adoptive t-cell | WO2019178207A1 | Memorial Sloan Kettering Cancer Center |
| Recombinant poxviruses for cancer immunotherapy | WO2024097448A1 | Memorial Sloan Kettering Cancer Center |
| Anti-CTLA-4 Antibodies and Methods of use of the same | AR104810A1 | Agenus Inc. |
| Uso de composicao MVA?E3L e veiculo ou Diluente Farmaceuticamente aceitavel | BR112017022134B1 | Memorial Sloan Kettering Cancer Center |
| | | |

**Supplemental Figures**

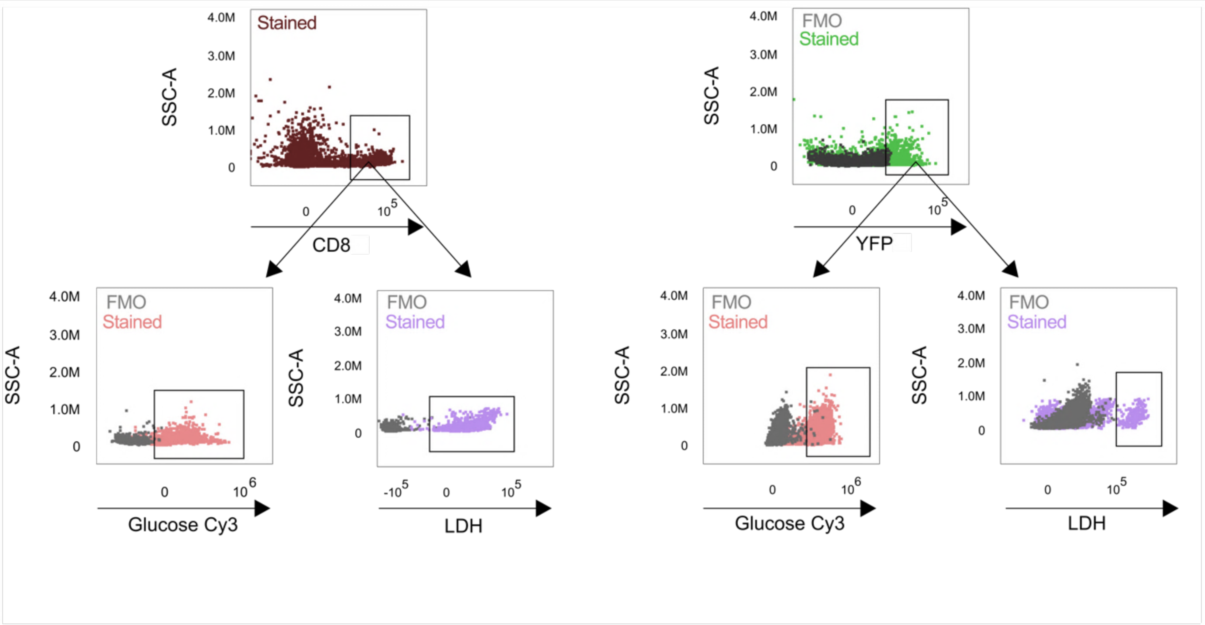


**Supplemental Figure 1. Gating strategy for Glucose Cy3 and LDH in YFP+ B16 tumor cells and CD8+ T cells.** Flow cytometry dot plots for Glucose-Cy3 and intracellular LDH staining compared to unstained or isotype controls (FMO, gray) for tumor- infiltrating CD8+ T cells and YFP+ B16 tumor cells from established B16-YFP murine tumors (corresponding to Main Figure 1F).

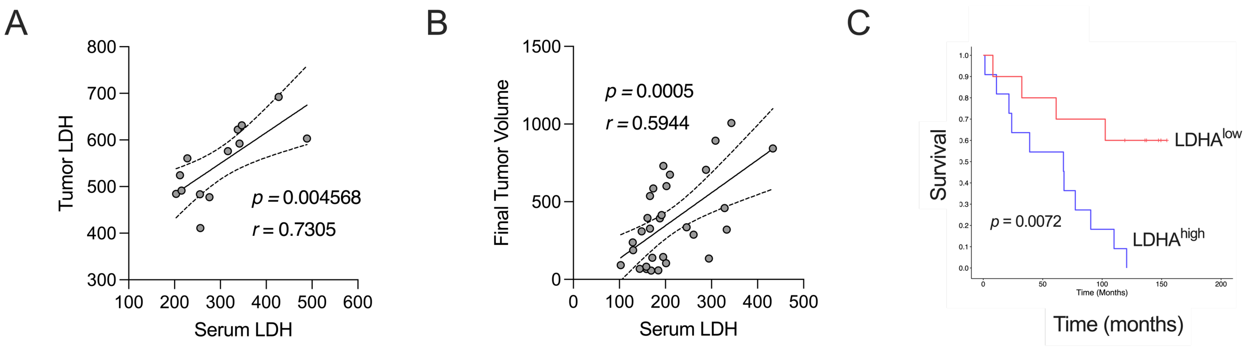


**Supplemental Figure 2. LDH correlates with poor prognosis.** (**A-B**) Correlations between B16 melanoma tumor LDH activity and serum LDH activity and between final B16 tumor burden and serum LDH activity quantified ex vivo from matched C57BL/6 mice 10 (**A**) and 20 (**B**) days of after tumor implantation, respectively. Data shown is representative of 3 independent experiments. Statistics produced by Spearman correlation tests in GraphPad. (**C**) Overall survival of melanoma patients stratified by tumor LDHA RNA expression. Samples were defined as LDHAhigh or LDHAlow by expression of LDHA above or below the median LDHA value across all samples respectively (Source: Riaz et al. (57)). Statistics produced by unpaired T test with Welch's correction implemented in R.

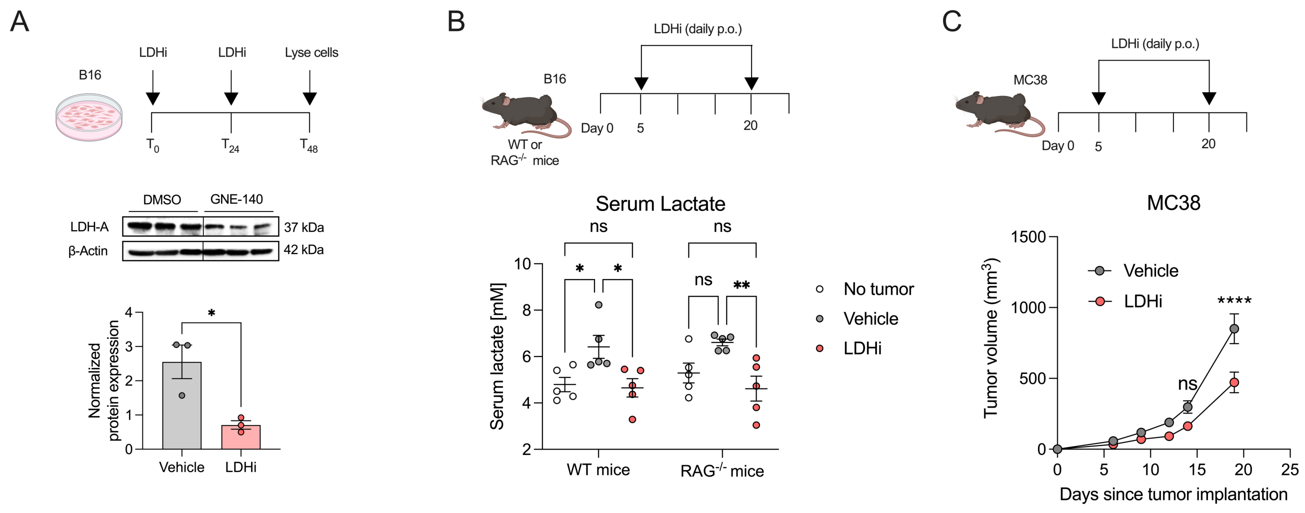


**Supplemental Figure 3. Effects of LDHi in mouse tumor models.** Corresponding to Main Figure 2. (**A**) Representative and densitometry quantification of western blot of LDHA and b-actin in whole cell lysates from B16 cells treated for 48hrs with 10 μM LDHi or vehicle control (DMSO). (**B**) Quantified serum lactate from the indicated B16-bearing mouse strains 1h after the last treatment with LDHi (100mg/kg) or control vehicle as indicated in the schema. (**C**) MC38 tumor growth curves upon treatment with LDHi (100 mg/kg) or control vehicle as indicated in the schema (n = 5/group). Data shown are one representative experiment of 3 independent experiments. All statistics produced by (**A**) Wilcoxon Rank Sum Test or (**B-C**) Two-way ANOVA with Bonferroni's multiple comparisons test implemented in GraphPad Prism in which *, $P<0.05$; **, $P<0.01$; ***, $P<0.001$; ****, $P<0.0001$. Data are mean +/– SEM.

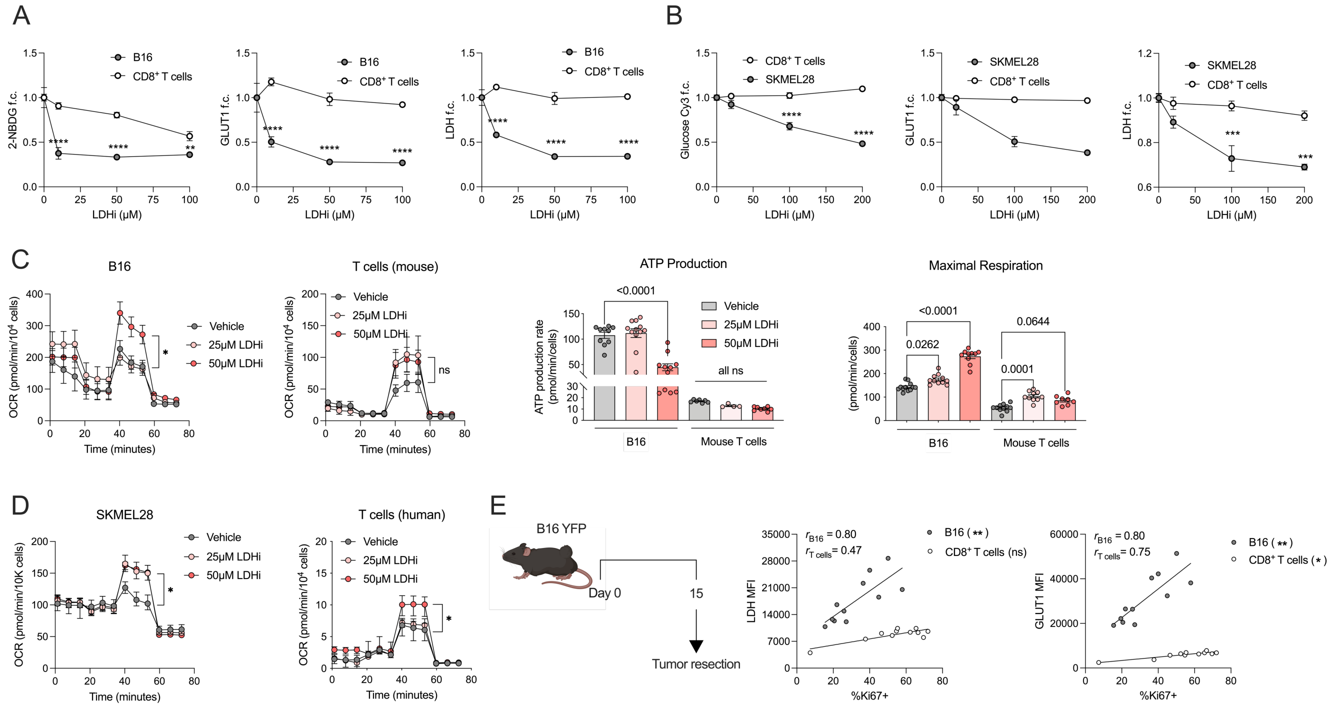


**Supplemental Figure 4. Tumor cells display more glycolytic sensitivity to LDH inhibition than immune cells. Corresponding to Main Figure 3.** (**A-B**) Fold-change values corresponding to data in Figure 3A-C, F-H. (**C**) Oxygen consumption rates (OCR), ATP production, and maximal respiration of B16 cells and activated mouse $CD8^+$ T cells treated with the indicated concentrations of LDHi or vehicle *in vitro,* normalized by cell number. Mouse T cells were treated with LDHi 24h post-aCD3/aCD28 activation and analyzed 24h later. (**D**) Oxygen consumption rates of SK-MEL-28 cells and activated human $CD8^+$ T cells treated with the indicated concentrations of LDHi or vehicle *in vitro*, normalized by cell number. Human T cells were treated with LDHi 48h post-aCD3/aCD28 activation and analyzed 24h later. (**E**) Correlations between LDH or GLUT1 expression (MFI) and %Ki67+ cells (gated based on the isotype control staining) in $CD8^+$ T cells and B16 $YFP^+$ tumor cells quantified by flow cytometry from B16 tumors from untreated mice. Data shown are one representative experiment of 3 independent experiments. All statistics produced by (**A-B**) Wilcoxon Rank Sum Test, (**E**) Pearson Correlation, and (**C-D**) Two-way ANOVA with Bonferroni's multiple comparisons test implemented in GraphPad Prism in which *, $P<0.05$; **, $P<0.01$; ***, $P<0.001$; ****, $P<0.0001$. Data are mean +/– SEM.

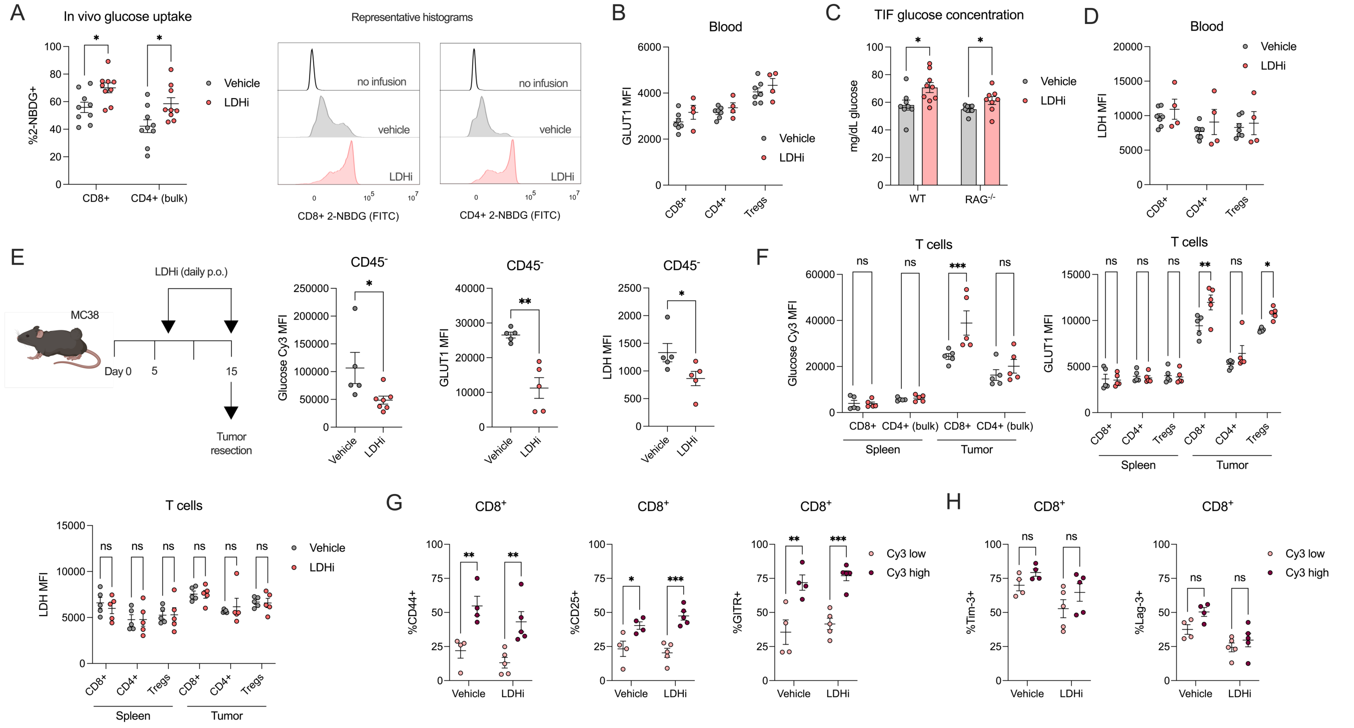


**Supplemental Figure 5. LDHi boosts glucose uptake of intratumoral T cells with no bioactivity in blood cells.** Corresponding to Main Figure 4. **(A-D)** Wild-type B16-bearing mice were treated with LDHi (100 mg/kg) or vehicle control for 10 days (n = 10/group) and (**A**) % 2-NBDG+ tumor-infiltrating T cells; and **(B)** GLUT1 and (D) LDH MFI in T cells from whole blood were quantified by flow cytometry; (**C**) and glucose concentrations in tumor interstitial fluid were quantified by glucose meter. **(E-F)** Quantification of Glucose Cy3, GLUT1, and LDH MFI by flow cytometry in (E) CD45$^-$ cells and **(F)** CD8$^+$, CD4$^+$Foxp3$^-$, and CD4$^+$Foxp3$^+$ T cell subsets isolated from tumors or spleens from MC38 bearing mice treated with LDHi (100 mg/kg) or vehicle control as indicated in the schema (n = 5 biological replicates). (**G-H**) Quantified (**G**) markers of activation: %CD44+, %CD25+, %GITR+, and (**H**) markers of exhaustion: %Tim3+, and %Lag3+, on B16 tumor-infiltrating CD8+ T cells stratified by high or low Glucose Cy3 uptake. Data shown are one representative experiment of 1-3 independent experiments. All statistics produced by Wilcoxon Rank Sum Test implemented in GraphPad Prism in which *, $P$<0.05; **, $P$<0.01; ***, $P$<0.001; ****, $P$<0.0001. Data are mean +/– SEM.

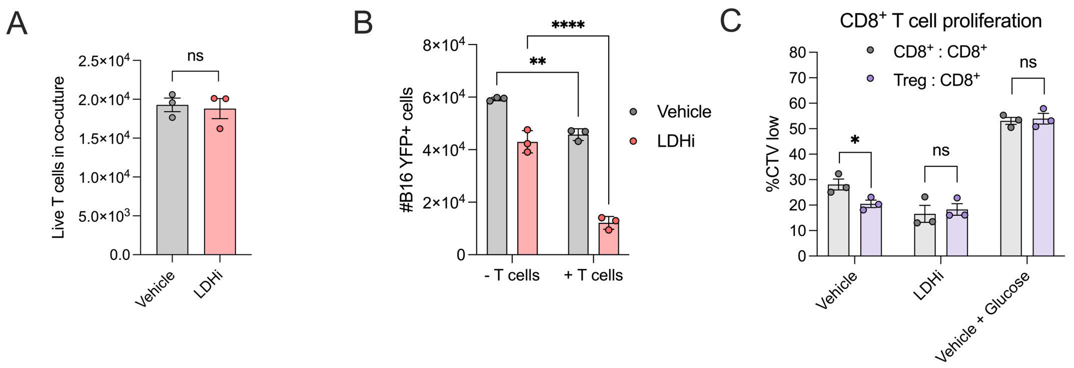


**Supplemental Figure 6. LDH inhibition improves anti-tumor T cell functions.** Corresponding to Main Figure 5. (**A**) Quantification of live $CD8^+$ T cells by flow cytometry after 48h killing assay co-culture, corresponding to experiment from Main Figure 5, C and D. (**B**) Raw data from Main Figure 5G: Quantification of live B16-YFP tumor cells from killing assay with $OVA_{257\text{-}264}$-pulsed live B16-YFP tumor cells and OVA-primed $CD8^+$ T cells from OT1 transgenic mice upon 48-hr co- culture in the presence of LDHi (as indicated in the schema from Main Figure 5A). Effector to target ratio, E:T = 2:1, co-cultured over 48h. (**C**) Raw data from Main Figure 5I: Quantification of $CD8^+$ T cell proliferation by CTV from in vitro Treg suppression assay with MACS column-sorted Tregs ($CD4^+CD25^+$ Regulatory T Cell Isolation Kit, mouse) co-cultured for 72hr with αCD3/αCD28 activated CTV-labeled syngeneic $CD8^+$ T cells and for 48hrs with the addition of conditioned media from B16 cells treated with 20 μM LDHi or vehicle, or fresh media containing 10 mM glucose. Data are from one representative experiment of 2-4 independent experiments (n = 3-4 biological replicates). All statistics produced by (**A**) Wilcoxon Rank Sum Test, and (**B-C**) Two-way ANOVA with Bonferroni's multiple comparisons test implemented in GraphPad Prism in which *, $P<0.05$; **, $P<0.01$; ***, $P<0.001$; ****, $P<0.0001$. Data are mean +/– SEM.

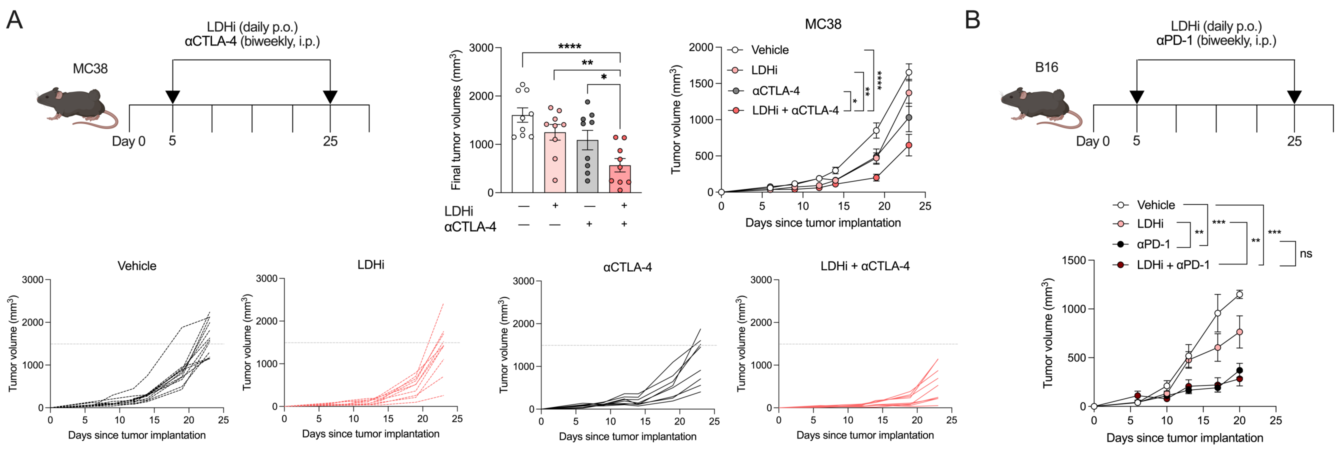


**Supplemental Figure 7. Anti-tumor activity of LDHi with ICB in vivo.** Corresponding to Main Figure 6. (**A**) Final tumor volumes and averaged and individual tumor growth curves ($mm^3$) from MC38 tumor-bearing mice treated with 100 mg/kg LDHi and/or CTLA-4 blockade (9D9, IgG2b) or control vehicle/IgG as indicated in the schema (n = 8-10 mice/group). (**B**) Tumor volumes ($mm^3$) from B16 tumor-bearing mice treated with early (day 5) PD-1 blockade (RPM1-14, IgG2b) and/or 100 mg/kg LDHi or control vehicle/IgG as indicated in the schema (n = 10-15 mice/group). Data are from one representative experiment of 2 independent experiments. All statistics produced by Wilcoxon Rank Sum Test or 2-way ANOVA implemented in GraphPad Prism in which *, $P<0.05$; **, $P<0.01$; ***, $P<0.001$; ****, $P<0.0001$. Data are mean +/– SEM.

**Table S1. Flow cytometry antibody details.**

| Antibodies | Vendor | Cat No. | Clone |
|---|---|---|---|
| Zombie NIR | eBioscience | 423105 | N/A |
| BUV395 Rat Anti-Mouse CD45 | BD Biosciences | 564279 | 30-F11 |
| BUV563 Rat Anti-Mouse CD62L | BD Biosciences | 741230 | MEL-14 |
| BUV615 Rat Anti-Mouse CD8a | BD Biosciences | 613004 | 53-6.7 |
| BUV737 Hamster Anti-Mouse CD11c | BD Biosciences | 612796 | HL3 |
| BUV805 Hamster Anti-Mouse TCR β Chain | BD Biosciences | 748405 | H57-597 |
| Brilliant Violet 421 anti-T-bet | BioLegend | 644816 | 4B10 |
| Pacific Blue anti-mouse H-2Kb | BioLegend | 116514 | AF6-88.5 |
| Pacific Blue anti-mouse H-2Kd | BioLegend | 116616 | SF1-1.1 |
| BV480 Rat Anti-Mouse CD274 (PD-L1) | BD Biosciences | 746275 | MIH5 |
| Brilliant Violet 510 anti-mouse/human CD11b | BioLegend | 101263 | M1/70 |
| Pacific Orange anti-mouse F4/80 | Invitrogen | MF48030 | BM8 |
| Brilliant Violet 570 anti-mouse Ly-6C | BioLegend | 128029 | HK1.4 |
| Super Bright 645 anti-mouse CD19 | Invitrogen | 64-0193-82 | eBio1D3 (1D3) |
| Super Bright 702 anti-mouse CD86 (B7-2) | Invitrogen | 67-0862-82 | GL1 |
| BV750 Rat Anti-Mouse CD357 (GITR) | BD Biosciences | 747402 | DTA-1 |
| Brilliant Violet 785 anti-mouse CD279 (PD-1) | BioLegend | 135225 | 29F.1A12 |
| Alexa Fluor 488 anti-mouse CD80 | BioLegend | 104716 | 16-10A1 |
| Spark Blue 550 anti-mouse I-A/I-E | BioLegend | 107662 | M5/114.15.2 |
| PerCP anti-mouse Ly-6G | BioLegend | 127654 | 1A8 |
| PerCP/Cyanine5.5 anti-mouse CD206 (MMR) | BioLegend | 141715 | C068C2 |
| PerCP-eFluor 710 anti-mouse FOXP3 | Invitrogen | 46-5773-82 | FJK-16s |
| PE/Dazzle 594 anti-mouse CD134 (OX-40) | BioLegend | 119418 | OX-86 |
| PE-Cyanine5 anti-mouse EOMES | Invitrogen | 15-4875-82 | Dan11mag |
| PE-Cyanine7 anti-mouse CD152 (CTLA-4) | Invitrogen | 25-1522-82 | UC10-4B9 |
| APC anti-mouse/human Granzyme B | Invitrogen | GRB05 | GB11 |
| Spark NIR 685 anti-mouse CD25 | BioLegend | 102070 | PC61.5 |
| Alexa Fluor 700 anti-mouse CD44 | Invitrogen | 56-0441-82 | IM7 |
| APC-eFluor 780 anti-mouse Ki-67 | Invitrogen | 47-5698-82 | SolA15 |

| | | | |
|---|---|---|---|
| APC/Fire 810 anti-mouse CD4 | BioLegend | 100480 | GK1.5 |
| BUV661 Hamster Anti-Mouse CD154 (CD40L) | BD Biosciences | 741499 | MR1 |
| BUV496 Anti-Mouse H-2Ld/H-2Db | BD Biosciences | 749923 | 28-14-8 |
| Alexa Fluor 647 anti-glucose transporter (GLUT1) | Abcam | Ab195020 | EPR3915 |
| PE anti-lactate dehydrogenase (LDH) | Abcam | Ab210445 | EP1563Y |
