# Supplementary material for: Pharmacologic LDH inhibition redirects intratumoral glucose uptake and improves antitumor immunity in solid tumor models
Source: J Clin Invest. 2024 Sep 3;134(17):e177606. doi: 10.1172/JCI177606 (PMC11364391; doi:10.1172/JCI177606)
Supplement: Unedited blot and gel images [file jci-134-177606-s245.pdf]

Full unedited gels for Supplemental Figure 3A*:

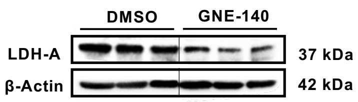

LDH-A

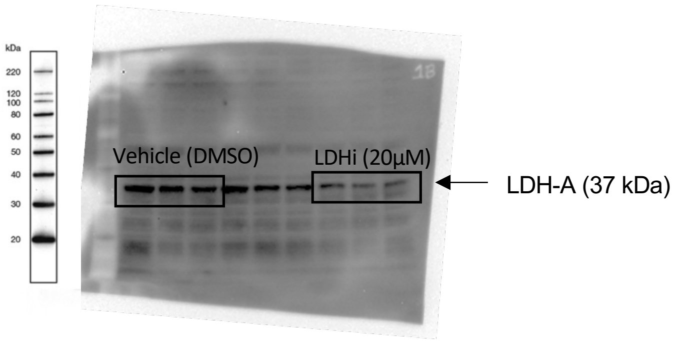

β-Actin

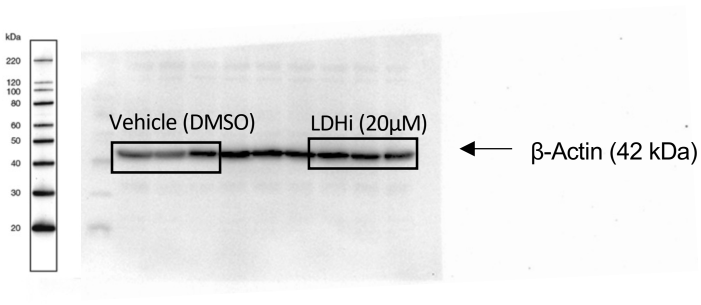

*Note: lanes 4-6 represent a drug treatment condition that is irrelevant to the current manuscript
